# RGS6 suppresses TGF-β-induced epithelial–mesenchymal transition in non-small cell lung cancers via a novel mechanism dependent on its interaction with SMAD4

**DOI:** 10.1038/s41419-022-05093-0

**Published:** 2022-07-28

**Authors:** Zhao Wang, Jun Chen, Shengjie Wang, Zelong Sun, Zhe Lei, Hong-Tao Zhang, Jie Huang

**Affiliations:** 1 Soochow University Laboratory of Cancer Molecular Genetics, Suzhou Medical College of Soochow University, Suzhou, Jiangsu 215123 China; 2 Department of Genetics, School of Biology and Basic Medical Sciences, Suzhou Medical College of Soochow University, Suzhou, Jiangsu 215123 China; 3 Department of Thoracic Surgery, The First Affiliated Hospital of Soochow University, Suzhou Medical College of Soochow University, Suzhou, Jiangsu 215006 China; 4 Department of Basic Medicine, Kangda College of Nanjing Medical University, Lianyungang, 222000 China; 5 Suzhou Key Laboratory for Molecular Cancer Genetics, Suzhou, Jiangsu 215123 China

**Keywords:** Cell signalling, Metastasis

## Abstract

Regulator of G-protein signaling 6 (RGS6) is a newly discovered tumor suppressor that has been shown to be protective in development of various cancers such as breast cancer and bladder cancer. But the mechanisms underlying these tumor-suppressing functions of RGS6 are not fully understood. Here, we discover a novel function of RGS6 in suppressing TGF-β-induced epithelial–mesenchymal transition (EMT) of non-small cell lung cancer (NSCLC) cells and in vivo NSCLC metastasis. Using both bioinformatics and experimental tools, we showed that RGS6 was downregulated in lung cancer tissues compared to noncancerous counterparts, and low expression of RGS6 was associated with poor survival of lung cancer patients. Overexpression of RGS6 suppressed TGF-β-induced EMT in vitro and TGF-β-promoted metastasis in vivo, by impairing gene expression of downstream effectors induced by the canonical TGF-β-SMAD signaling. The ability of RGS6 to suppress TGF-β-SMAD-mediated gene expression relied on its binding to SMAD4 to prevent complex formation between SMAD4 and SMAD2/3, but independent of its regulation of the G-protein signaling. Interaction between RGS6 and SMAD4 caused less nuclear entry of p-SMAD3 and SMAD4, resulting in inefficient SMAD3-mediated gene expression. Taken together, our findings reveal a novel and noncanonical role of RGS6 in regulation of TGF-β-induced EMT and metastasis of NSCLC and identify RGS6 as a prognostic marker and a potential novel target for NSCLC therapy.

## Introduction

Lung cancer has been ranked as the number one cancer in both case numbers and death rate in past several years [1, 2]. Non-small lung cancer (NSCLC) accounts for 85% of lung cancer cases and is known for high risk of metastasis and low survival rate [3]. Metastasis is a complex multi-step process that is believed to account for over 90% of cancer-caused mortality [4, 5]. The epithelial–mesenchymal transition (EMT), an essential event in embryonic development and tissue repair, has been implicated as a key process in initiation of metastasis [6]. Among the pathways known to induce EMT, transforming growth factor beta (TGF-β) signaling has been demonstrated as one of the most common and essential pathways underlying metastasis of various cancer types including NSCLC [7–9]. After being activated by TGF-β, TGF receptor type I (TβRI) can act through the canonical SMAD-dependent pathway, or SMAD-independent pathways (e.g. through PI3K-Akt pathway or MAPK pathway) to induce EMT [10, 11].

RGS6 belongs to the RGS protein superfamily that is well known to act as GTPase-activating proteins toward Gα subunits to negatively regulate heterotrimeric G-protein signaling [12–14]. Although aberrant activation of G-protein signaling has been found in development and progression of various cancer types [15, 16], understanding of the roles of RGS proteins in cancers remains relatively poor. Some members of the RGS protein family, including RGS6, have been shown to exhibit tumor-suppressing effects [17]. As a key regulator to shut down the G-protein signaling, RGS6 has been shown to be involved in many important metabolic processes, such as cardiac parasympathetic control and so on [18–21]. A role of RGS6 in preventing tumorigenesis was first suggested by a finding that a single nucleotide polymorphism (SNP), which increases RGS6 gene expression up to 3 folds, is correlated with low risk of developing bladder cancer [22]. Since then, more and more anti-tumor actions of RGS6 have been discovered. RGS6 induces cell cycle arrest and apoptosis of cancer cells [23]. RGS6 also induces ROS (reactive oxygen species) formation to activate the ATM-p53 pathway, an action required by Doxorubicin, one of the most widely used chemotherapeutic drugs, to induce cancer cell death [24]. Besides its ability to induce apoptosis, RGS6 inhibits oncogenic transformation induced by Ras, one of the most important oncogenic proteins mutated in over 30% human cancers, by promoting TIP60-mediated degradation of another oncogenic protein DNMT1 [25]. Moreover, downregulation of RGS6 expression has been found in various cancer tissues, including breast cancer [26], bladder cancer [27], colorectal cancer [28] and pancreatic cancer [29]. These studies strongly support a role of RGS6 as a tumor suppressor. However, RGS6 expression has been found to be greatly upregulated in certain types of ovarian cancer cell lines [30]. Although this study does not report any biological function of the upregulated RGS6 transcripts in these ovarian cancer cells, and there is no evidence indicating that RGS6 may exhibit either pro- or anti-tumor actions depending on cancer types, these differential expression profiles of RGS6 in different types of cancer cells render thorough investigations to elucidate the molecular mechanisms underlying the tumor-suppressing function of RGS6 necessary for application of RGS6 in development of new anti-cancer therapy. Here, we first investigated the function of RGS6 in lung cancer development and particularly, in cancer metastasis.

## Materials and methods

### Cell culture and transfection

The human NSCLC cells A549 and H1299 were obtained from the Cell Bank of Chinese Academy of Sciences (Shanghai, China). Cells were cultured using standard protocol. Detailed procedures are listed in Supplementary Materials and Methods.

### Tissue samples

Paired fresh frozen human lung cancer tissues and adjacent noncancerous lung tissues were collected after informed consent from patients in the First Affiliated Hospital of Soochow University. All patients had been diagnosed with lung cancer followed by histological and pathological characteristics according to the Revised International System for Staging Lung Cancer, and patients received neither chemotherapy nor radiotherapy before tissue sampling. Clinical characteristics of the patients are detailed in Supplementary Table S1. This study was approved by the Ethics Committee of Soochow University.

### RNA extraction, cDNA synthesis, and quantitative real-time PCR (qRT-PCR)

RNA extraction, cDNA synthesis and qRT-PCR were performed using standard protocols. Detailed information is listed in Supplementary Materials and Methods. Primers are listed in Supplementary Table S6.

### Western blotting assay and nuclear fractionation

Western blotting was carried out as described previously [31]. Information of antibodies is listed in Supplementary Materials and Methods. Uncropped WB blots used in all Figures are included in Supplementary file_Uncropped blots.

### Construction of HA-tagged RGS6 and Flag-tagged SMAD4 expression vectors

GFP-tagged RGS6 (full length and deletion mutants) expressing vectors were kindly provided by Prof. Fisher Rory. Detailed procedures of plasmids construction are listed in Supplementary Materials and Methods.

### Generation of stable cell lines overexpressing RGS6

To generate A549 and H1299 cell lines stably overexpressing RGS6, the full-length human RGS6 gene was sub-cloned into a pLenti-GIII-CMV-GFP-2A-Puro overexpression vector (Applied Biological Materials Inc., Zhenjiang, China) with restriction endonucleases *Nhe*I and *Xba*I. Detailed procedures are listed in Supplementary Materials and Methods.

### Production of RGS6 Knockout cells by the CRISPR/Cas9 system

Two single guide RNAs (sgRNA) sequences (Supplementary Table S5) targeting RGS6 exon 3 and 5 were designed using the online CRISPR design tool (https://zlab.bio/guide-design-resources). Detailed procedures are listed in Supplementary Materials and Methods.

### Co-immunoprecipitation (co-IP)

Co-immunoprecipitation was performed as described previously [32]. Detailed procedures are listed in Supplementary Materials and Methods.

### Immunohistochemistry (IHC)

Immunohistochemistry was performed on the sections (5 μm thickness) from the lung TMA. Detailed procedures are listed in Supplementary Materials and Methods.

### Migration and invasion assays

Migration and invasion assays were performed as described before [33]. Detailed procedures are listed in Supplementary Materials and Methods.

### Wound-healing assay

Migration and invasion assays were performed as described before [33]. Detailed procedures are listed in Supplementary Materials and Methods.

### Immunofluorescence staining

Immunofluorescent staining was performed as described previously [33]. Detailed procedures are listed in Supplementary Materials and Methods.

### Dual-luciferase reporter assay

Dual-luciferase reporter assay was performed as described previously [33]. Detailed procedures are listed in Supplementary Materials and Methods.

### Tumor xenografts and metastasis models

NSCLC xenografts and metastasis models were established as previously described [34]. Detailed procedures are listed in Supplementary Materials and Methods.

### Statistical analysis

Data are expressed as the mean ± SD and were compared using Students *t*-test for paired. Pearson’s correlation coefficient test or paired *t*-test (2-tailed) were performed to evaluate the significance of the data from patient samples. Differences with *p* < 0.05 were considered significant. All statistical analyses were conducted using GraphPad Prism 7 software (GraphPad, San Diego, CA, USA).

## Results

### RGS6 expression is downregulated in human NSCLC tissues

**Fig. 1:**
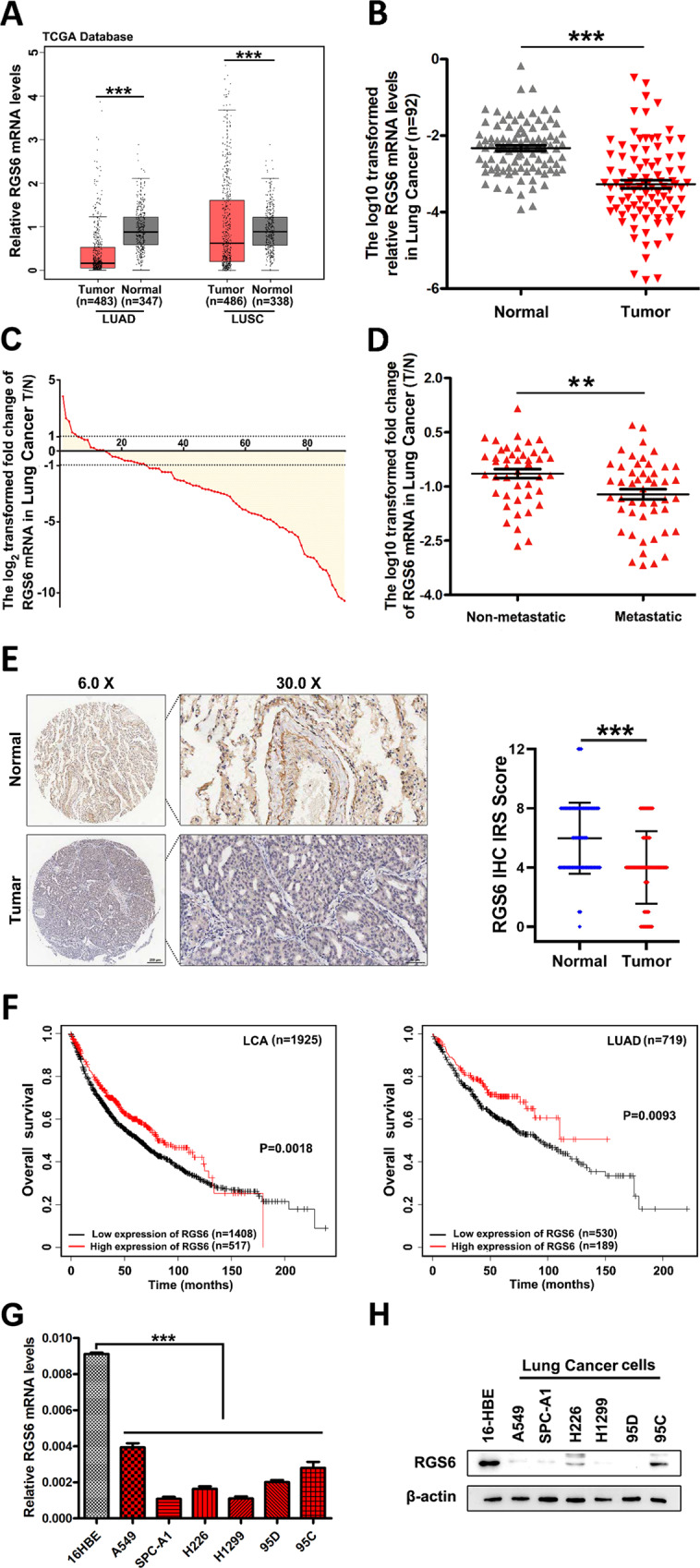
RGS6 expression is downregulated in human NSCLC tissues. **A** RGS6 mRNA expression in cancer tissues compared with the non-cancer counterparts from the LUAD and LUSC patients from TCGA. LUAD, lung adenocarcinoma; LUSC, lung squamous cell carcinoma (***p < 0.001). **B** qRT-PCR analysis of RGS6 mRNA levels in 92 human lung cancer tissues and paired noncancerous lung tissues. Mean values are indicted by solid bars, and values are expressed as mean ± SEM. T, tumor tissues; N, paired noncancerous lung tissues. **C** Relative expression of RGS6 mRNA in 92 paired lung cancer tissues. Y-axis represents the log2 transformed fold change of T/N expression ratios of RGS6 mRNA. The number of each specimen is shown below X-axis. **D** Relative expression (T/N) of RGS6 in non-metastatic (*n* = 44) and metastatic (*n* = 48) lung cancer tissues. Non-metastatic tissues were from lung cancer patients without any metastasis and metastatic tissues were from lung cancer patients with lymph node metastasis or distant metastasis (**p < 0.01). **E** RGS6 protein levels in lung cancer tissues and paired adjacent normal tissues were examined by IHC in tissues from 75 lung cancer patients. Left panel, representative IHC images. Right panel, RGS6 protein levels in tumor and normal tissues were quantified and shown as the IHC immunoreactivity score (IRS) (***p < 0.001). **F** Correlation between low RGS6 expression level and poor prognosis in lung cancer patients. LCA, lung carcinoma. Kaplan–Meier plots were generated using Kaplan–Meier Plotter (http://www.kmplot.com) (**p < 0.01). **G** RGS6 mRNA levels, **H** RGS6 protein levels in human lung epithelial cells (16-HBE) and six NSCLC cell lines (five adenocarcinoma cell lines: A549, SPC-A1, H1299, 95D and 95 C; as well as one squamous cell carcinoma cell line: H226). Each experiment was performed in triplicates (***p < 0.001).

To determine the role of RGS6 in the development of NSCLC, we first analyzed The Cancer Genome Atlas (TCGA) dataset and discovered that the mRNA levels of RGS6 were significantly reduced in tumor tissues from LUAD (lung adenocarcinoma) and LUSC (lung squamous cell carcinoma) patients compared to normal counterparts (Fig. 1A). To validate the clinical significance of these data, we next examined RGS6 mRNA levels in 92 human lung cancer tissues (including 78 NSCLC cases) and paired adjacent noncancerous tissues (Supplementary Table S1). Consistent with the result of TCGA analysis (Fig. 1A), a pattern of downregulation of RGS6 expression was indicated by the lower overall mRNA levels of RGS6 in tumor tissues compared with noncancerous lung tissues (Fig. 1B), and by reduced T/N (Tumor/Normal, i.e RGS6 levels in tumor tissues/RGS6 levels in paired normal tissues) ratios of RGS6 levels found in 84.7% (78 out of 92) examined human lung cancer cases (Fig. 1C). To further confirm downregulation of RGS6 expression in lung cancer tissues, comparison of RGS6 protein levels between lung cancer tissues and paired adjacent noncancerous lung tissues by IHC was performed with tissue samples from 75 LUAD patients (Supplementary Tables S3, S4). Like the mRNA levels, the overall protein levels of RGS6 in tumor tissues were significantly reduced compared to normal tissues (Fig. 1E). Surprisingly, in tumor tissues, RGS6 expression was downregulated in both the tumor cells and the stromal cells. Since IHC staining of all 75 paired tumor and normal tissues was performed on the same chip (Supplementary Fig. 1), the difference of RGS6 immunostaining between the tumor tissues and the normal tissues could not be caused by differential staining of these tissues. The mechanism regulating RGS6 expression during cancer development remains unclear. Based on the dramatic changes of the histological structures in the tumor tissue (Fig. 1E), one possibility is that changes in the micro-environments in the tumor tissue induced by tumor cells result in downregulation of RGS6 not only in the tumor cells but also in the stromal cells.

When we examined the correlation between RGS6 expression and important clinical parameters, we found that metastatic lung cancer tissues possessed even lower levels of RGS6 compared to non-metastatic tumor tissues (Fig. 1D), whereas no significant correlation was found between RGS6 expression and other clinical parameters examined (Supplementary Table S2). Since metastasis of NSCLC is often associated with poor prognosis, we subsequently investigated the correlation between RGS6 expression and the overall survival of lung cancer patients utilizing the Kaplan–Meier Plotter (http://www.kmplot.com). The survival analysis showed that low expression of RGS6 was significantly associated with poor survival in LCA patients and LUAD patients (Fig. 1F), but not in LUSC patients (Supplementary Fig. 2A). Whether or not this difference between LUAD and LUSC patients reflects different levels of RGS6 expression between LUAD tumor tissues and LUSC tumor tissues is worthy of further study. TCGA dataset analysis showed that the mRNA levels of RGS6 might be greater in LUSC patients compared to LUAD patients (Fig. 1A). However this trend was not shown when we analyzed mRNA levels of RGS6 in tumor tissues from NSCLC patients (Supplementary Fig. 2B, C), probably due to limited number of samples. Collectively, our data showed that RGS6 expression was downregulated in lung cancer tissues, especially in metastatic lung cancer tissues, and low RGS6 expression was associated with poor survival in LUAD patients, suggesting that RGS6 may play a role in suppressing NSCLC metastasis.

### Loss of RGS6 boots TGF-β-induced EMT of NSCLC cells

**Fig. 2:**
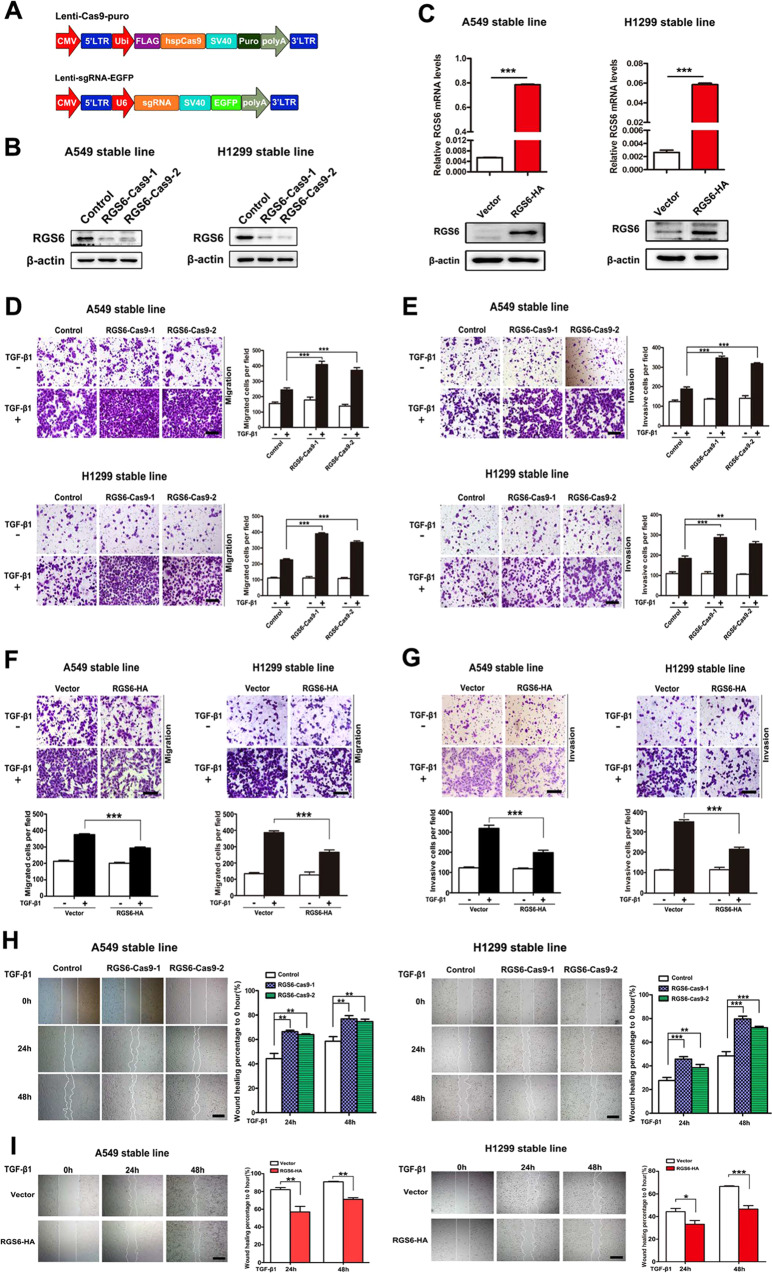
Loss of RGS6 boots TGF-β-induced EMT events in NSCLC cells, whereas overexpression of RGS6 represses TGF-β-induced EMT events NSCLC cells. **A** Schematic diagram of the CRISPR/Cas9 lentiviral system. **B** Confirmation of generation of two RGS6-KO NSCLC cell lines by western blotting. **C** Generation of NSCLC cell lines stably overexpressing RGS6. qRT-PCR (upper) and western blotting (lower) were used to confirm RGS6 overexpression. **D**, **F** In the presence or absence of TGF-β, indicated cells were allowed to migrate through 8-μm pores on the bottom of the transwell insert for 24 h. Migrated cells were stained and counted in at least three microscopic fields. Representative images (**D** left, **F** upper) and quantitated data (**D** right, **F** lower) are shown. Scale bar, 100 μm (***p < 0.001). **E**, **G** In the presence or absence of TGF-β, indicated cells were allowed to invade through the Matrigel-coated transwell insert for 24 h. Invasive cells were stained and counted in at least three microscopic fields. Representative images (**E** left, **G** upper) and quantitated data (**E** right, **G** lower) are shown. Scale bar, 100 μm. (**p < 0.01, ***p < 0.001) **H**, **I** Wound-healing assay was performed to evaluate TGF-β-induced (5 ng/ml) cell migration of indicated cells. The wound edges were photographed at the indicated time points after wounding. Each experiment was performed in triplicates. Representative images (left) and representative quantitated data (right) are shown. Scale bar, 200 μm. (**p < 0.01, ***p < 0.001).

Consistent with the downregulated pattern of RGS6 expression in lung tumor tissues, both RGS6 mRNA levels (Fig. 1G) and protein levels (Fig. 1H) were significantly lower in NSCLC cell lines examined. To examine the hypothesis that RGS6 plays a negative role in NSCLC metastasis, we designed two sgRNAs that specifically target RGS6 exons (Fig. 2A, Supplementary Fig. 3A and Supplementary Table S5). Two clones of RGS6 knockdown (KD) cell lines: RGS6-Cas9-1 and RGS6-Cas9-2, were constructed with two different NSCLCL cell lines: A549 and H1299, using the CRISPR/Cas9-mediated homologous recombination technique. Confirmation of mutation was carried out by PCR and T7 endonuclease I assay (Supplementary Fig. 3B, C). RGS6 protein levels in these KD cell lines were examined by western blotting to verity the cleavage efficiency (Fig. 2B).

EMT is one of the key steps in cancer metastasis and TGF-β-induced signaling pathways are among the best studied mechanisms underlying cancerous EMT events, such as cell migration and cell invasion. We then examined TGF-β-induced EMT of RGS6-KD (RGS6-Cas9) NSCLC cells. Migration assay and invasion assay were used to evaluate TGF-β-induced cell migration and invasion of control and RGS6-Cas9 NSCLC cell lines. Both TGF-β-induced cell migration (Fig. 2D) and invasion (Fig. 2E) were enhanced in RGS6-Cas9 cell lines compared to control cell lines. Cell migration induced by TGF-β was also examined using wound-healing assay. As shown in Fig. 2H, the wound-healing rates of RGS6-Cas9 cell lines were significantly increased compared with control cell lines.

### Overexpression of RGS6 represses TGF-β-induced EMT of NSCLC cells

To further confirm the role of RGS6 in inhibiting NSCLC metastasis, we next tested the effect of RGS6 overexpression on TGF-β-induced EMT of NSCLC cells. Stable cell lines overexpressing RGS6 in A549 and H1299 cells were constructed and the overexpression effect was verified on both mRNA and protein levels (Fig. 2C). Migration assay and invasion assay were then performed and showed that TGF-β-induced migration (Fig. 2F) and invasion (Fig. 2G) were both significantly suppressed in RGS6-HA stable cell lines compared with control cell lines. The rates of wound healing of RGS6-HA stable cell lines were also significantly lowered compared with control cell lines, confirming a suppressive effect of RGS6 on TGF-β-induced NSCLC cell migration (Fig. 2I). Together, these results exhibited that TGF-β-induced EMT were enhanced in NSCLC cells lacking RGS6, but suppressed in NSCLC cells overexpressing RGS6, suggesting an inhibitory effect of RGS6 on TGF-β-induced NSCLC EMT.

### RGS6 attenuates TGF-β-promoted metastasis of NSCLC cells in vivo

**Fig. 3:**
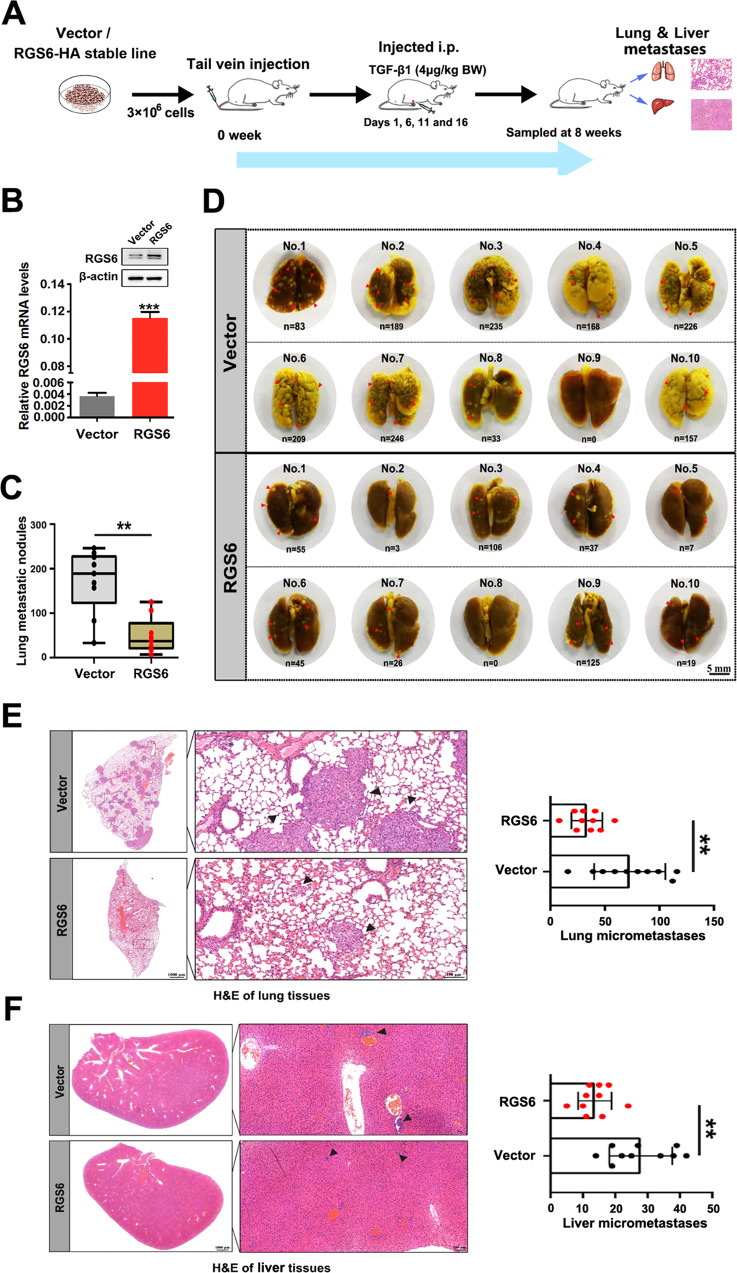
RGS6 attenuates TGF-β-promoted metastasis of NSCLC cells in vivo. **A** Schematic diagram of process of generation of in vivo TGF-β-promoted metastasis model. **B** Western blotting was performed to confirm RGS6 overexpression. **C** Quantification of macroscopically observable metastatic nodules in lung tissues from the RGS6-overexpressed group and control group. (**p < 0.01) **D** Pictures of lung tissues from each animal in the two groups. Some obvious macroscopically observable metastatic nodules are pointed by red arrow heads. **E** Left, representative images of lung tissue sections from animals in the two groups. Micrometastases are pointed by arrow heads. Right, quantification of micrometastases in lung tissues from animals in the two groups. (**p < 0.01) **F** Left, representative images of liver tissue sections from animals in the two groups. Micrometastases are pointed by arrow heads. Right, quantification of micrometastases in liver tissues from animals in the two groups. (**p < 0.01).

Our next step was to generate a tumor xenografts and metastasis model to further examine the negative effect of RGS6 on NSCLC metastasis. The process of generation of the TGF-β-promoted in vivo metastasis model is summarize in Fig. 3A. Confirmation of RGS6 expression in RGS6-HA stable NSCLC cells is shown in Fig. 3B. 8 weeks after tail-vein injection of RGS6-HA or control A549 cells, lung and liver tissues were harvested and analyzed as described in Methods. Pictures of the lung tissues from each animal of the RGS6-HA group and the control group are shown in Fig. 3D. Examples of macroscopically observable metastatic nodules (MOMN) are pointed out by red arrow heads. The number of these MOMNs were counted for each animal of the two groups. The overall numbers of MOMNs in mice injected with RGS6-HA A549 cells were significantly lower compared to mice injected with control A549 cells (Fig. 3C). The lung and liver tissues were then subjected to histological analysis for detection of micrometastases in these tissues. Sample pictures of lung tissue sections (Fig. 3E left) and liver tissue sections (Fig. 3F left) for each group are shown. Numbers of micrometastases in all tissue slides of the two groups were counted and summarized in Fig. 3E right and Fig. 3F right. Not only the overall numbers of micrometastases were significantly lower in both lung and liver tissues from the RGS6-HA group compare to the control group, but also the size of micrometastases in these tissues from animals in the RGS6-HA group appeared to be much smaller. Consistent with our in vitro study, loss of RGS6 promoted TGF-β-induced NSCLC metastasis in vivo, indicated by significantly higher numbers of MOMNs in lung tissues from mice injected with RGS6-Cas9-1 or RGS6-Cas9-2 NSCLC cells (Supplementary Fig. 4B, C), as well as by significantly more micrometastases in lung (Supplementary Fig. 4D) and liver (Supplementary Fig. 4E) tissues from these mice. All together, these studies provide solid evidence that RGS6 not only suppresses TGF-β-induced EMT of NSCLC cells in vitro, but also attenuates TGF-β-promoted NSCLC metastasis in vivo.

### RGS6 interacts with SMAD4 and prevents complex formation between R-SMADs and SMAD4

**Fig. 4:**
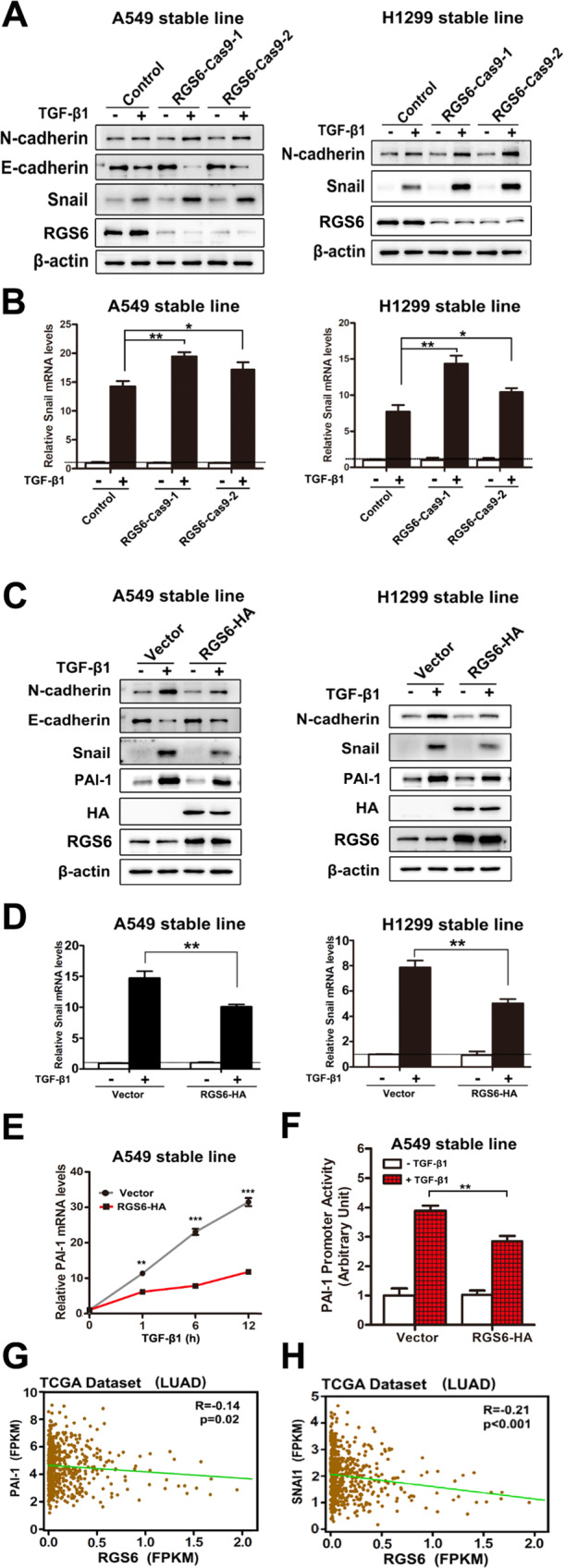
RGS6 blocked activation of downstream effectors of TGF-β-induced pro-EMT signaling. **A** After being serum starved for 24 h, RGS6-Cas9 NSCLC cells and control cells were treated with or without TGF-β (5 ng/ml) for 24 h. Western blotting was performed to detect expression of EMT-related markers and effectors E-cadherin, N-cadherin and Snail. Noted that there is no E-cadherin expression in H1299 cells. **B** RGS6-Cas9 NSCLC cells and control cells were treated with or without TGF-β1 (5 ng/ml) for 2 h, and subjected to qRT-PCR for detection of Snail mRNA levels (*p < 0.05, **p < 0.01). **C** After being serum starved for 24 h, RGS6-HA stable NSCLC cells and control cells were treated with or without TGF-β (5 ng/ml) for 24 h. Western blotting was performed to detect expression of E-cadherin, N-cadherin and Snail. **D** RGS6-HA stable NSCLC cells and control cells were treated with or without TGF-β1 (5 ng/ml) for 2 h, and subjected to qRT-PCR for detection of Snail mRNA levels (**p < 0.01). **E** RGS6-HA A549 cells and control cells were treated with TGF-β (5 ng/ml) for indicated time periods, and subjected to qRT-PCR for detection of PAI-1 mRNA levels (**p < 0.01, ***p < 0.001). **F** The PAI-1 promoter constructs were transiently transfected into RGS6-HA and control A549 cells, and the relative luciferase activities were determined using the Dual-Luciferase reporter assay system after being treated with TGF-β (5 ng/ml) for 24 h (**p < 0.01). **G** RGS6 level is negatively correlated with PAI-1 expression in LUAD patients from TCGA dataset. *X* and *Y* axes represent levels of RGS6 and PAI-1 mRNA, respectively. **H** RGS6 level is negatively correlated with SNAI1 gene expression in LUAD patients from TCGA dataset. X and *Y* axes represent levels of RGS6 and SNAI1 mRNA, respectively.

We next examined the effect of RGS6 on TGF-β-induced EMT markers. In RGS6-Cas9 NSCLC cells, TGF-β-induced expression of Snail was enhanced as examined on both mRNA and protein levels (Fig. 4A, B). Consistently, upregulation of N-cadherin protein and downregulation of E-cadherin protein were also enhanced in RGS6-Cas9 cells (Fig. 4A). In contract, both the mRNA level and protein level of Snail induced by TGF-β were reduced in RGS6-HA stable cells (Fig. 4C, D), as well as attenuation of TGF-β-induced upregulation of N-cadherin and downregulation of E-cadherin (Fig. 4C). We also examined another well-known TGF-β-induced EMT marker: PAI-1. TGF-β-induced upregulation of PAI-1 mRNA level was significantly blocked in RGS6-HA stable A549 cells (Fig. 4E). TGF-β-induced increase of PAI-1 promoter activity was significantly attenuated in RGS6-HA stable cells examined by the luciferase reporter assay (Fig. 4F), confirming that RGS6 blocks TGF-β-induced PAI-1 gene expression. RGS6 overexpression impaired TGF-β-induced upregulation of PAI-1 protein level in NSCLC cells with little effect on basal level of PAI-1 (Fig. 4C), suggesting that the effect of RGS6 on PAI-1 promoter activity is TGF-β-dependent. Consistently, we discovered that expression levels of PAI-1 and SNAI1 genes were inversely correlated with the levels of RGS6 in LUAD patients by analyzing TCGA dataset (Fig. 4G, H), suggesting that RGS6 suppresses TGF-β-induced activation of Snail and PAI-1 in both NSCLC cells and tissues.

**Fig. 5:**
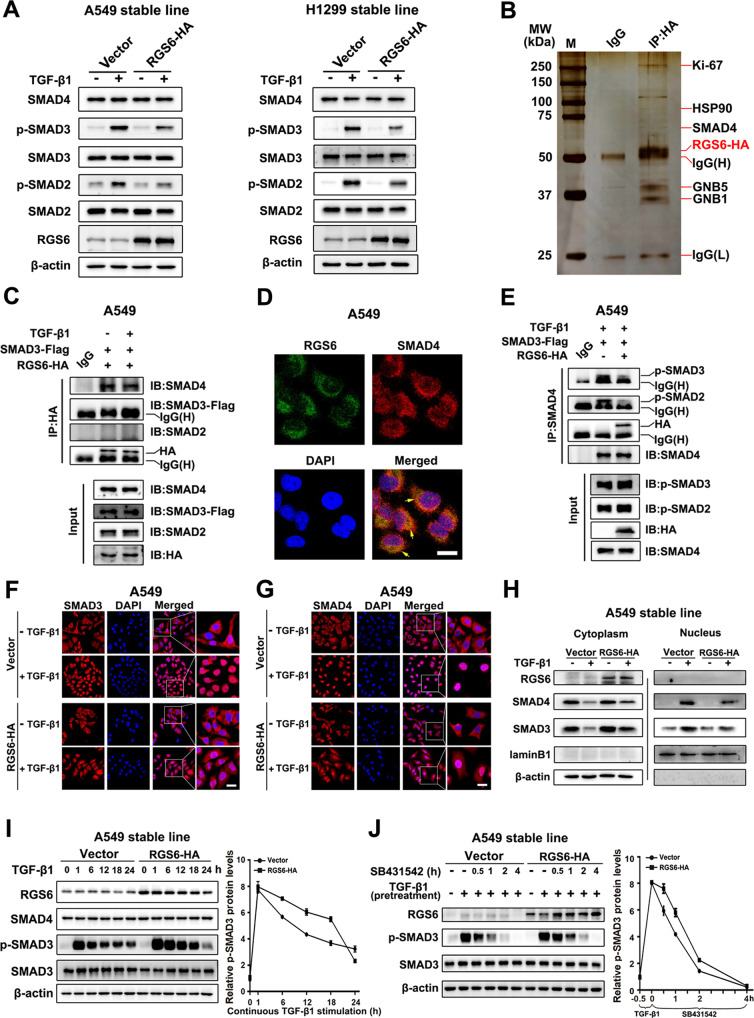
RGS6 interacts with SMAD4 and prevents complex formation between SMAD4 and phosphorylated-R-SMADs, slowing down nuclear entry of SMAD3 and SMAD4. **A** RGS6-HA stable and control NSCLC cells were treated with TGF-β (5 ng/ml) for 24 h. Levels of phosphorylated R-SMADs and total levels of R-SMADs and SMAD4 were examined by western blotting. **B** Sample western blot with silver stain showing RGS6-associated proteins identified in mass spectrometry analysis. **C** HA-tagged RGS6 and Flag-tagged SMAD3 were co-transfected in A549 cells, cells were then treated with or without TGF-β (5 ng/ml) for 1 hour before subjected to immunoprecipitation with anti-HA antibody and probed for indicated proteins. **D** A549 cells were fixed and incubated with the indicated primary antibody simultaneously, then stained with a FITC-conjugated anti-mouse IgG (green, for RGS6) and a Cy3-conjugated anti-rabbit IgG (red, for SMAD4). Finally, nuclei were counterstained with DAPI (blue). Scale bar, 10 μm. **E** HA-tagged RGS6 and Flag-tagged SMAD3 were co-transfected in A549 cells, cells were then treated with or without TGF-β (5 ng/ml) for 1 hour before subjected to immunoprecipitation with anti-SMAD4 antibody and probed for indicated proteins. **F**, **G** RGS6-HA stable and control A549 cells were treated with or without TGF-β (5 ng/ml) for 2 h, followed by ICC staining for SMAD3 (**F**) and SMAD4 (**G**). DAPI staining was used for visualization of the nuclei. Scale bar, 20 μm. **H** RGS6-HA stable and control A549 cells were treated with or without TGF-β (5 ng/ml) for 1 hour followed by nuclear and cytoplasmic fractionation. Extracts of both fractions were subjected to immunoblotting for detection of indicated proteins. Lamin B1 and β-actin were used as internal controls for nuclear and cytoplasmic extracts, respectively. **I** RGS6-HA stable and control A549 cells were treated with TGF-β (5 ng/ml) for the indicated time periods, and subjected to western blotting for detection of indicated proteins. Left: a representative western blotting. Right, quantification of intensity of p-SMAD3 relative to total SMAD3 in three independent experiments using ImageJ software. **J** RGS6-HA stable and control A549 were treated with TGF-β (5 ng/ml) for 30 min, followed by TGF-β washout and simultaneous addition of 5 mM SB431542. After treatment of SB431542 for indicated time periods, cell lysates were collected and blotted for indicated proteins. Left: a representative western blotting. Right, quantification of intensity of p-SMAD3 relative to total SMAD3 in three independent experiments using ImageJ software.

To explore the molecular mechanism by which RGS6 inhibits activation of the downstream effectors of TGF-β pro-EMT signaling, we started with the canonical TGF-β-SMAD pathway. In RGS6-HA stable NSCLC cells, TGF-β-induced phosphorylation of SMAD2 and SMAD3 were both attenuated at 24 h after TGF-β treatment, while no change on the total levels of these two proteins and SMAD4 (Fig. 5A). Since SMAD4 had been identified as an RGS6 binding partner in a mass spectrometry we carried out before (Fig. 5B and Supplementary Fig. 5), we went on to test whether interaction between RGS6 and SMAD4 is related to RGS6-mediated attenuation of SMAD2/3 phosphorylation. As shown in Fig. 5C, RGS6 was co-immunoprecipitated with SMAD4 in the absence of TGF-β, but not with SMAD2 or SMAD3. TGF-β treatment did not affect the binding affinity of RGS6 with SMAD4 or R-SMADs. Without TGF-β treatment, binding between RGS6 and SMAD4 was also confirmed by co-localization of these two proteins in the cytoplasm using immunocytochemistry (Fig. 5D). In another co-IP experiment, we found that the presence of RGS6 significantly reduced the amount of phosphorylated SMAD2 and phosphorylated SMAD3 pulled down by anti-SMAD4 antibody, indicating that the interaction between RGS6 and SMAD4 preventing complex formation between SMAD4 and phosphorylated SMAD2/3 (Fig. 5E).

Complex formation between SMAD4 and phosphor-SMAD2/3 is believed to facilitate translocation of activated SMAD2/3 into the nucleus where they can trigger expression of their target genes. Therefore, preventing formation of complex between SMAD4 and phosphor-SMAD2/3 by RGS6 should result in retaining of R-SMADs in the cytoplasm. As indeed, we found that RGS6 overexpression partially blocked TGF-β-induced nuclear translocation of both SMAD3 and SMAD4 as shown by western blotting (Fig. 5H). This result was also confirmed by immunocytochemistry (ICC). Upon TGF-β stimulation, there was more cytoplasmic ICC staining of SMAD3 (Fig. 5F) and SMAD4 (Fig. 5G) in RGS6-HA stable cells compared with control cells. Although a previous study reports nuclear translocations of RGS6 under stress conditions [35], we did not observe trafficking of RGS6 into the nuclei of NSCLC cells during TGF-β treatment (Fig. 5H). Therefore, the inhibitory effects of RGS6 on transcription of pro-EMT effectors downstream of the TGF-β-SMAD axis (Fig. 4F) is least likely to be caused by direct actions of RGS6 on the promoter of these genes inside the nucleus.

To explore whether impaired nuclear translocation of R-SMADs and SMAD4 results in attenuation of R-SMADs phosphorylation observed in RGS6-HA stable cells (Fig. 5A), we investigated the kinetics of TGF-β-induced phosphorylation of SMAD3 in NSCLC cells. In the presence of TGF-β, phosphorylation of SMAD3 peaked at 1 hr followed by gradually weakening overtime in a 24 h period in both control and RGS6-HA stable cells (Fig. 5I). Surprisingly, although same as observed earlier (Fig. 5A), SMAD3 phosphorylation at the 24 hr time point was reduced in RGS6-HA cells, at earlier time points examined in this time course study, levels of SMAD3 phosphorylation were all much higher in RGS6-HA cells compared with control cells (Fig. 5I). Since it is acknowledged that the nucleus is the major site for dephosphorylation of R-SMADs upon TGF-β treatment [36, 37], this unexpected pattern of TGF-β-induced SMAD3 phosphorylation in RGS6-HA cells is consistent with our earlier observations that a greater portion of phosphorylated SMAD3 was retained in the cytoplasm in these cells compared with control cells (Fig. 5F, H). Therefore, the cytoplasmic pool of phosphorylated SMAD3 was protected from being dephosphorylated, resulting in a higher level of phosphorylated SMAD3 in RGS6-HA cells.

To better investigate dephosphorylation of SMAD3 after TGF-β treatment, we pretreated A549 cells with TGF-β for 30 mins to generate a pool of p-SMAD3, followed with treatment of a TβRI inhibitor SB431542 to prevent re-phosphorylation of dephosphorylated SMAD3. Dephosphorylation of the existing pool of p-SMAD3 finished within 4 h in both control and RGS6-HA cells (Fig. 5J). Consistent with the earlier time course study (Fig. 5I), the rate of SMAD3 dephosphorylation in RGS6-HA cells was slower than that in control cells (Fig. 5J). This result not only confirms that retaining of p-SMAD3 in the cytoplasm in RGS6-HA cells protects p-SMAD3 from dephosphorylation in these cells, but also suggests that RGS6 slows down dephosphorylation of SMAD3 without affecting phosphorylation of SMAD3 by TGF-β. Interestingly, there was a dramatic decline of p-SMAD3 level between 18–24 hrs after TGF-β treatment in RGS6-HA cells, suggesting that although slowing down p-SMAD dephosphorylation in the early stage of TGF-β application, RGS6 promotes dephosphorylation of p-SMAD3 in the late stage of TGF-β treatment to shut down the TGF-β-SMAD signaling (Fig. 5I). However, RGS6-mediated accelerated dephosphorylation of p-SMAD3 in the 18–24 h time window is unlikely to be the mechanism by which RGS6 suppresses TGF-β-induced upregulation of EMT effectors, Snail and PAI-1, since this suppression was observed as early as at 2 and 1 hr time point, respectively (Fig. 4D, E). Previous investigations have shown that SMAD4 is important for interaction between R-SMADs and co-activators, such as p300/CBP, for triggering R-SMAD-mediated gene expression [38–40]. It is more likely that RGS6 prevents complex formation between SMAD4 and R-SMADs and nuclear entry of SMAD4, resulting in poor association between R-SMADs with co-activators and subsequent inefficiency of R-SMAD-mediated gene expression. Consistent with this hypothesis, the kinetics of SMAD3 dephosphorylation after TGF-β treatment in SMAD4-KO (SMAD4-Cas9) A549 cells (Supplementary Fig. 6) were very similar to those in RGS6-HA cells (Fig. 5I, J). Therefore, we next examined whether RGS6-mediated suppression of TGF-β-induced EMT requires SMAD4.

### RGS6 suppresses the TGF-β-SMAD pro-EMT signaling and TGF-β-induced EMT via a SMAD4-dependent mechanism

**Fig. 6:**
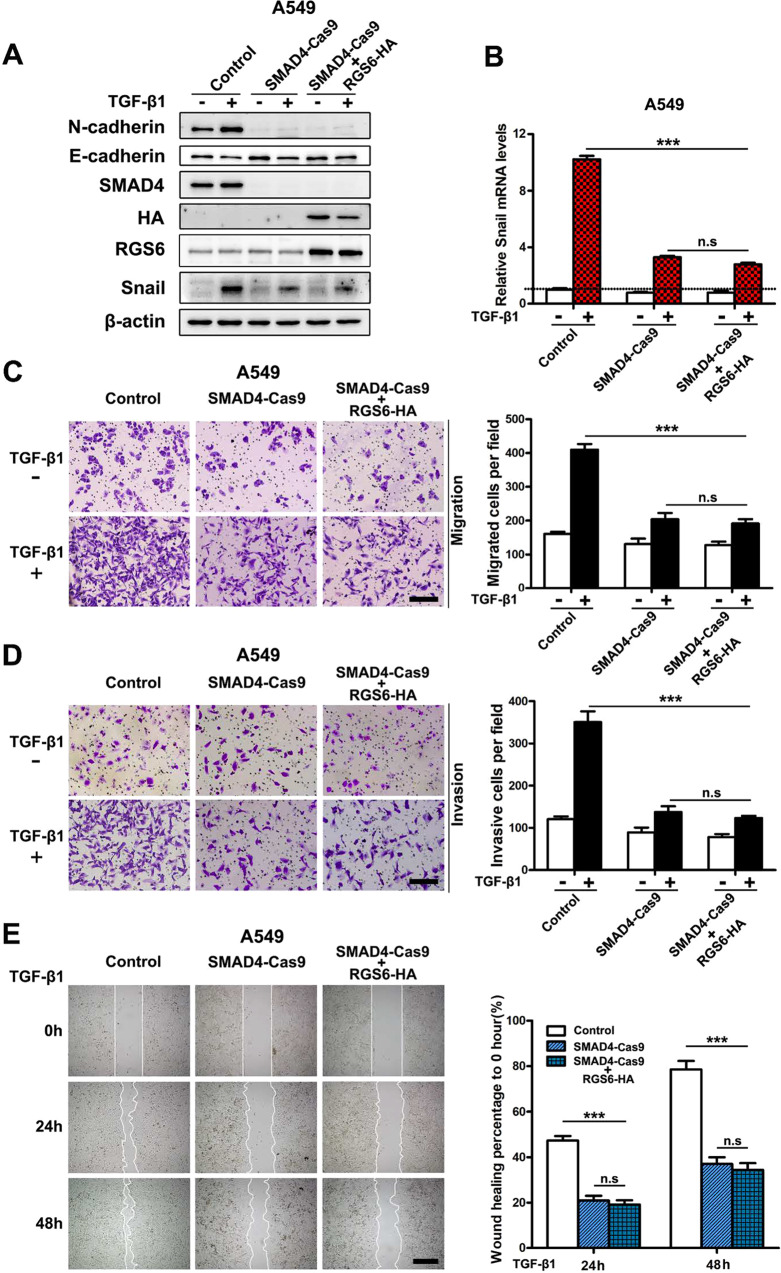
RGS6 suppresses TGF-β-induced pro-EMT signaling and EMT events via a SMAD4-dependent mechanism. We examined the effect of RGS6 overexpression on TGF-β-induced pro-EMT signaling and EMT events in SMAD4-KD cells. If RGS6 can act through a mechanism not involving SMAD4 to suppress TGF-β-induced EMT, overexpressing RGS6 should cause a greater inhibition on TGF-β-induced EMT in SMAD4-KD cells. **A** SMAD4-Cas9 cells were transfected with RGS6 or control vector. Cells were then treated with or without TGF-β (5 ng/ml) for 24 h. Cell lysates were collected and subjected to immunoblotting for indicated proteins. **B** SMAD4-Cas9 cells were transfected with RGS6 or control vector. Cells were then treated with or without TGF-β (5 ng/ml) for 2 h and subjected to qRT-PCR for detection of Snail mRNA level (***p < 0.001). **C**, **D** SMAD4-Cas9 cells were transfected with RGS6 or control vector and subjected to migration assay (**C**) and invasion assay (**D**). Left, representative images. Right, quantification of cell migration (**C**) and cell invasion (**D**). Scale bar, 100 μm (***p < 0.001). **E** SMAD4-Cas9 cells were transfected with RGS6 or control vector and subjected to wound-healing assay. Left, representative images. Right, quantification of rate of wound healting. Scale bar, 200 μm (***p < 0.001).

To evaluate whether the inhibitory effect of RGS6 on the TGF-β-SMAD pro-EMT signaling relies on its interaction with SMAD4, we took advantage of the SMAD-KD A549 cells. If RGS6 can act through a mechanism not involving SMAD4 to suppress TGF-β-induced NSCLC EMT, overexpression of RGS6 in SMAD4-Cas9 cells should result in a combined and greater inhibition of TGF-β-induced NSCLC EMT events. We overexpressed RGS6 in SMAD4-Cas9 A549 cells and examined symbolic molecular changes in TGF-β-induced EMT. TGF-β-induced downregulation of E-cadherin, upregulation of N-cadherin and Snail were all blocked in SMAD4-Cas9 cells (Fig. 6A, B). Overexpression of RGS6 had no additive effect on these molecular changes in SMAD4-Cas9 cells (Fig. 6A, B). We also examined TGF-β-induced NSCLC cells migration and invasion of SMAD4-Cas9 cells. Similarly, overexpression of RGS6 had no additive effect on inhibition of TGF-β-induced NSCLC cells migration (Fig. 6C, E) or invasion (Fig. 6D) in SMAD4-Cas9 cells. Together, these results suggest that RGS6-mediated suppression of TGF-β-induced EMT is SMAD4-dependent.

**Fig. 7:**
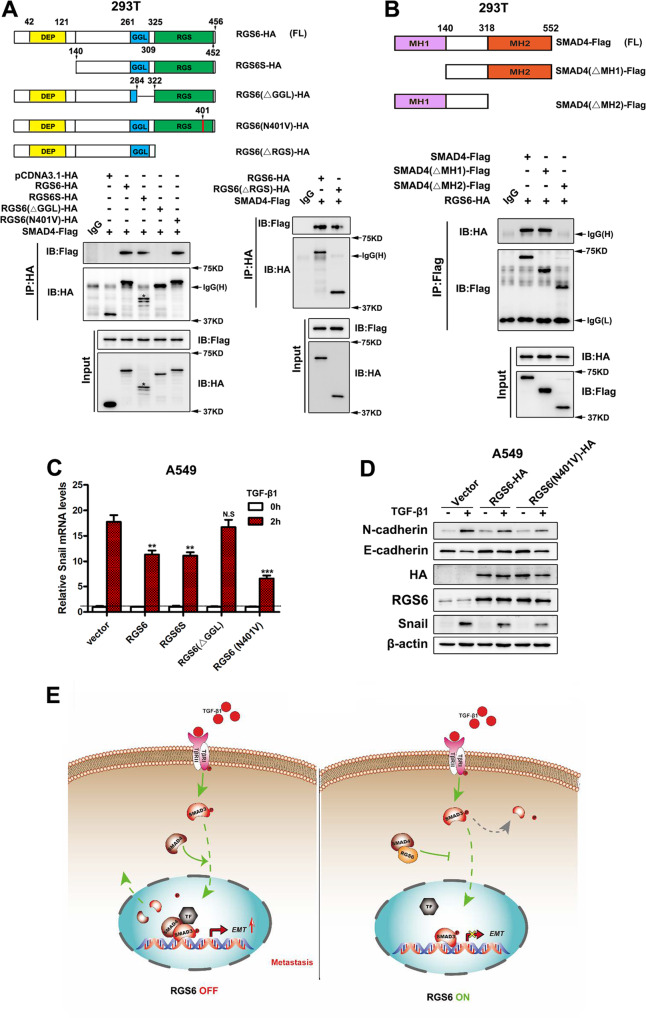
Interaction between RGS6 and SMAD4 is mediated via association between the GGL domain and the MH2 domain. **A** Upper, schematic illustration of RGS6 mutants. Lower, 293 T cells were co-transfected with Flag-tagged SMAD4 and various HA-tagged RGS6 mutants. Cell lysates were collected and subjected to IP with anti-HA antibody and probed for indicated proteins. **B** Upper, schematic illustration of SMAD4 mutants. Lower, 293 T cells were co-transfected with HA-tagged RGS6 and various Flag-tagged SMAD4 mutants. Cell lysates were collected and subjected to IP with anti-Flag antibody and probed for indicated proteins. **C** A549 cells transfected with different RGS6 mutants were treated with or without TGF-β (5 ng/ml) for 2 h. Cell lysates were collected and subjected to qRT-PCR for detection of Snail mRNA level (**p < 0.01, ***p < 0.001). **D** A549 cells transfected with different RGS6 mutants were treated with or without TGF-β (5 ng/ml) for 24 h. Cell lysates were collected and subjected to immunoblotting for indicated proteins. **E** Schematic diagram summarizing the effects of RGS6 on the TGF-β-SMAD pro-EMT signaling. Upon TGF-β stimulation, complex formation between SMAD4 and phosphorylated R-SMAD, such as SMAD3, facilitates nuclear translocation of p-SMAD3 into the nucleus, where SMAD4 further promotes association between p-SMAD3 and co-activators (TF) to ensure full efficiency of SMAD3-mediated gene expression, followed by dephosphorylation of SMAD3 and recycling of SMAD3 and SMAD4 back into the cytoplasm. RGS6 interacts with SMAD4 and prevents complex formation between SMAD4 and p-SMAD3. Interaction between RGS6 and SMAD4 causes cytoplasmic retaining of p-SMAD3 and SMAD4, leading to less p-SMAD3 in the nucleus and poor association between p-SMAD3 and co-activators, finally resulting in inefficient SMAD3-mediated gene expression.

Besides the core RGS domain, RGS6 has other two main structural domains: the DEP (Dishevelled, Egl-10, Pleckstrin homology) domain and the GGL (Gγ subunit like) domain [14]. To clarify which structural domain mediates the interaction between RGS6 and SMAD4, we performed co-IP experiment using different mutants of RGS6, each of which missing one particular domain (Fig. 7A upper). Only RGS6(ΔGGL), the mutant RGS6 that lacks the GGL domain, failed to be co-IPed with SMAD4 (Fig. 7A lower), indicating that the GGL domain is required for interaction between RGS6 and SMAD4. SMAD4 has two MH (Mad homology) domains that mediate interaction between SMAD4 and R-SMADs. Using different mutants of SMAD4, each of which missing one particular MH domain (Fig. 7B upper), we found that the MH2 domain was required for interaction between SMAD4 and RGS6 (Fig. 7B lower). Our data indicated that complex formation between RGS6 and SMAD4 is mediated by association between the GGL domain of RGS6 and the MH2 domain of SMAD4.

To further confirm the significance of the GGL domain in this negative regulation of the TGF-β-SMAD signaling by RGS6, we examined how these mutant forms of RGS6 affected downstream effectors of the TGF-β-SMAD signaling. As shown in Fig. 7C, overexpression of RGS6(ΔGGL) had no effect on TGF-β-induced upregulation of Snail mRNA level, while other mutant forms of RGS6 all significantly reduced TGF-β-induced Snail expression. We also examined whether the GAP activity of RGS6 plays a role in this novel anti-tumor action of RGS6. RGS6(N401V), a GAP activity-deficient mutant, interacted with SMAD4 (Fig. 7A lower) and attenuated TGF-β-induced changes in expression of EMT markers (Fig. 7C, D) as the full length RGS6 protein. All together, our work reveals a novel action of RGS6 in suppressing TGF-β-induced EMT by acting on the TGF-β-SMAD signaling. This action of RGS6 is independent of its GAP activity but relies on its interaction with SMAD4. RGS6-SMAD4 interaction blocks complex formation between SMAD4 and R-SMADs and results in suppression of TGF-β-induced gene expression via (1) slowing down nuclear entry of p-SMAD3; (2) preventing SMAD4 from facilitating association between SMAD3 and co-activators for efficient gene transcription (Fig. 7E).

## Discussion

Our study discovers a novel anti-tumor action of RGS6 in suppressing TGF-β-induced EMT of NSCLC cells. This novel action of RGS6 relies on its interaction with SMAD4, which prevents complex formation between SMAD4 and R-SMADs, resulting in retention of p-SMAD3 and SMAD4 in the cytoplasm. Translocation of the SMAD2/3/4 hetero-oligomer into the nucleus is essential for TGF-β-induced gene expression in many systems [41]. However, nuclear entry of SMAD2/3 appears not to rely solely on SMAD4 as the R-SMADs contain nuclear localization signals in their MH1 domains that can bring them into the nucleus without SMAD4 association [38, 42–44]. In our study, disrupting association between SMAD4 and SMAD3 by RGS6 partially blocked nuclear translocation of SMAD3 and did not prevent dephosphorylation of p-SMAD3 in the presence of TβRI inhibitor, suggesting that in NSCLC cells, nuclear entry of p-SMAD3 in response to TGF-β treatment occurs via both SMAD4-dependent and -independent mechanisms. As a native nuclear protein, the cytoplasmic localization of SMAD4 is a result of active nuclear export and help from anchor proteins such as TRAP-1 [45–47]. Formation of the R-SMADs-SMAD4 oligomer may not be necessary for nuclear entry of R-SMADs in response to TGF-β treatment, but appears to be crucial for association of R-SMADs with co-activators p300/CBP and for full efficiency of R-SMADs-mediated gene expression [38–40]. Therefore, our study suggests that RGS6 suppresses TGF-β-induced EMT via following mechanisms: (1) slowing nuclear entry of p-SMAD3 to reduce the amount of p-SMAD3 in the nucleus; (2) preventing nuclear entry of SMAD4 resulting in inefficient SMAD3-mediated gene expression.

TGF-β can induce complicated responses in cancer progression, switching from tumor-suppressing by inhibiting cell proliferation at an early stage to pro-oncogenic by promoting EMT and angiogenesis at late stages [48, 49]. Although our study indicates that the inhibitory effects of RGS6 on TGF-β-induced EMT events in the in vitro system depends on its interaction with SMAD4 (Fig. 6), this novel tumor-suppressing action of RGS6 may not be the only one underlying its protective role against TGF-β-promoted in vivo NSCLC metastases (Fig. 3). Previous investigations show that RGS6 can prevent cell growth or induce apoptosis in cancer cell lines [23–25]. We also observed an inhibitory effect of RGS6 on cell growth and cell cycle progression of NSCLC cells (Supplementary Fig. 7). Considering the fact that many NSCLC cell lines are resistant to TGF-β-induced growth inhibition [50], it would be particularly interesting to systematically investigate whether RGS6 can help to restore response of NSCLC cells to TGF-β-induced growth inhibition or apoptosis and whether the ability of RGS6 to induce growth inhibition and apoptosis contributes to its function in suppressing TGF-β-induced metastases in vivo.

In the passing decade, a number of studies have suggested differential effects of RGS family members on angiogenesis, including both tumor and physiological angiogenesis. Downregulation of RGS4 [51], RGS5 [52] and RGS16 [53] is associated with enhanced angiogenesis, whereas downregulation of RGS3 [54] and RGS1 [55] impairs angiogenesis in respective systems. The finding that double mutant mice lacking both RGS6 and oxidative CaMKIIδ show embryonic defects in cardiac vasculogenesis implies a significant role of RGS6 in physiological angiogenesis [56]. Tumor angiogenesis is a very complicated process that differs significantly from physiological angiogenesis and can be tumor-type-dependent [57]. Our study suggests that NSCLC could be a useful system to investigate the role of RGS6 in tumor angiogenesis, which may have great impact on our understanding of the function of RGS6 in cancer development and its clinical application.

Upon TGF-β stimulation, TβRI activates R-SMADs through phosphorylation in the SXS motif of R-SMADs. The nuclear phosphatase PPM1A/PP2C has been implicated as the major phosphatase for R-SMADs that dephosphorylates phosphorylation of the SXS motif [36, 37]. PPM1A-mediated dephosphorylation of SMAD2/3 promotes dissociation between SMAD2/3 and SMAD4 and facilitate nuclear export of SMAD2/3 [37, 58]. The long-lasting high levels of SMAD3 phosphorylation within 1–18 h of TGF-β application in both control and RGS6-HA cells (Fig. 7D) suggest that: (1) dephosphorylated SMAD3 proteins were re-phosphorylated quickly after being exported out of the nucleus in the cytoplasm; (2) there was none or very little action of phosphatase toward SXS phosphorylation in the cytoplasm in the first 18 h of TGF-β treatment. An intriguing finding in this study is that there was a dramatic decline of p-SMAD3 level in RGS6-HA cells and SMAD4-KO cells between 18–24 h after TGF-β treatment, implying an RGS6-mediated upregulation of cytoplasmic phosphatase activity within that time window. Interesting, RGS12, another RGS family member, was recently shown to directly associate with and activate the phosphatase PTEN [59]. However, a link between RGS6 and phosphatase has never been found. It would be of great significance to uncover the mechanism by which RGS6 promotes cytoplasmic phosphatase activity towards R-SMADs to shut down the TGF-β-SMAD signaling in that particular time window after TGF-β treatment.

RGS6 is not the first RGS protein that was found to interact with a SMAD protein. Yau et al reported that RGS3 interacts with SMAD proteins to inhibit complex formation between SMAD3 and SMAD4, resulting in impaired TGF-β-induced gene transcription [60]. Different from our observation, this early study showed that RGS3 does not affect TGF-β-induced SMAD2 phosphorylation [60]. This difference could be due to following two reasons: (1) different types of cells were used (CHO cells vs NSCLC cells); (2) the time points were chosen to examine the level of SMAD phosphorylation. In the early study, SMAD2 phosphorylation was examined at 30 mins after TGF-β treatment [60]. At this time point in our time course study, TGF-β-induced SMAD3 phosphorylation hasn’t peaked yet and there was no obvious difference between the p-SMAD3 levels in RGS6-HA and control cells. More importantly, we examined subcellular localization of SMAD3 and SMAD4 in control and RGS6-HA stable cells after TGF-β treatment, which along with our time course studies of p-SMAD3 phosphorylation and dephosphorylation, providing more solid evidence supporting mechanisms by which RGS6 suppresses TGF-β-induced gene expression stated above. Our study identifies the GGL domain (254–319) as the SMAD-binding site of RGS6, a region adjacent to the C-terminal RGS domain. Interestingly, the region that mediates interaction between RGS3 and SMAD proteins was mapped to a structurally similar domain on RGS3: amino acids 240–379 just outside the C-terminal RGS domain. But the interactions between RGS proteins and SMAD proteins reported in these two studies are very different: RGS3 interacts with SMAD2, SMAD3 and SMAD4 [60], while RGS6 interacts only with SMAD4. One possible cause of this different binding affinity could be the difference in amino acid sequence of the two binding sites, as these two regions have no obvious homology in sequence. Another possible cause could be involvement of other structural domain(s) of the RGS protein. It has been reported previously that the GGL domain and the RGS domain of RGS6 can scaffold different proteins in the same complex [25]. Although our finding that RGS6 mutant lacking the RGS domain could bind SMAD4 as effectively as the full length RGS6 rules out the possibility that the RGS domain is involved in RGS6-SMAD4 binding, taking consideration the different binding affinity towards SMAD proteins between RGS3 and RGS6, the high homology in the MH2 domain among these three SMAD proteins, and the structural similarities between the two binding sites of RGS6 and RGS3, we can’t exclude the possibility that the RGS domain of RGS3 facilitates association between the binding site of RGS3 and the MH2 domain of SMAD proteins. Interestingly, there seems to be some indirect evidence favoring this hypothesis. Axin, a protein containing an RGS-homologous domain, is found to interact with SMAD3 through binding to the MH2 domain of SMAD3 and help anchoring SMAD3 to the cell membrane [61]. Although it was not examined in that study whether or not the RGS-homologous domain of Axin is involved in this interaction between Axin and SMAD3, it was shown in another report that the 1–183 region of Axin, which contains most of the RGS-homologous domain (89–216) [62], bound weakly to SMAD7 [63].

In summary, this work demonstrates a novel function of RGS6 in formation and metastasis of lung cancer. Both RGS6 mRNA and protein levels are downregulated in human lung cancer tissues compared to noncancerous counterparts. Low level of RGS6 is more prominent in metastatic lung cancer tissues and correlated with poor prognosis of lung cancer patients. Overexpression of RGS6 suppresses TGF-β-induced EMT of NSCLC cells in vitro and TGF-β-promoted metastasis of NSCLC cells in vivo. The ability of RGS6 to suppress TGF-β-SMAD axis and TGF-β-induced gene expression relies on its interaction with SMAD4 to interrupt complex formation between SMAD4 and R-SMADs and to interfere nuclear entry of SMAD4 and R-SMADs. This work not only provides new insights into our understanding of the dephosphorylation events in TGF-β signaling and expands our knowledge of anti-tumor signaling actions of RGS6, but also provide solid evidence supporting RGS6 as a prognostic marker and a potential novel target for NSCLC therapy.

## Supplementary information

Supplementary Figures and Legends

Reproducibility checklist

Supplementary Materials and Methods

Supplmentary Table S1

Supplmentary Table S2

Supplmentary Table S3

Supplmentary Table S4

Supplmentary Table S5

Supplmentary Table S6

Uncropped WB blots

## Data Availability

All data generated or analyzed during this study are available from the corresponding author on reasonable request.
