# Supplementary material for: RGS6 suppresses TGF-β-induced epithelial–mesenchymal transition in non-small cell lung cancers via a novel mechanism dependent on its interaction with SMAD4
Source: Cell Death Dis. 2022 Jul 28;13(7):656. doi: 10.1038/s41419-022-05093-0 (PMC9334288; doi:10.1038/s41419-022-05093-0)
Supplement: Supplementary file 1 — Supplementary Figures and Legends [file 41419_2022_5093_MOESM1_ESM.docx]

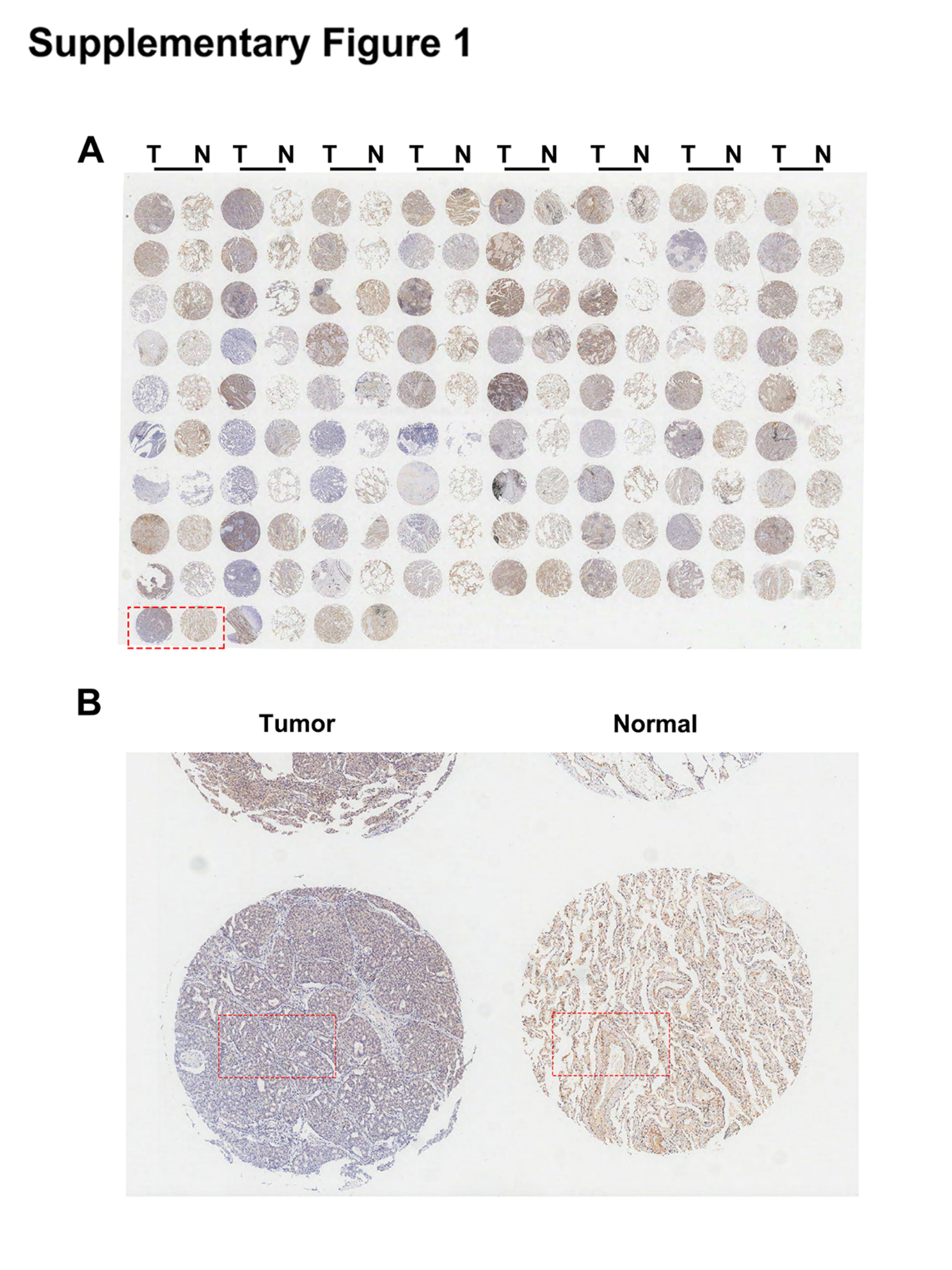


***Supplementary Figure 1. IHC staining for RGS6 in paired tumor and normal tissues from LUAD patients***

(**A**) A scan of the whole chip containing paired tumor and normal tissues from 75 LUAD patients is shown. The two tissues marked in the red box are the sample tissues chosen to be shown in Fig. 1E. (**B**) A magnified view of the sample tissues. The areas marked in red boxes are examined under a higher magnification in Fig. 1E.


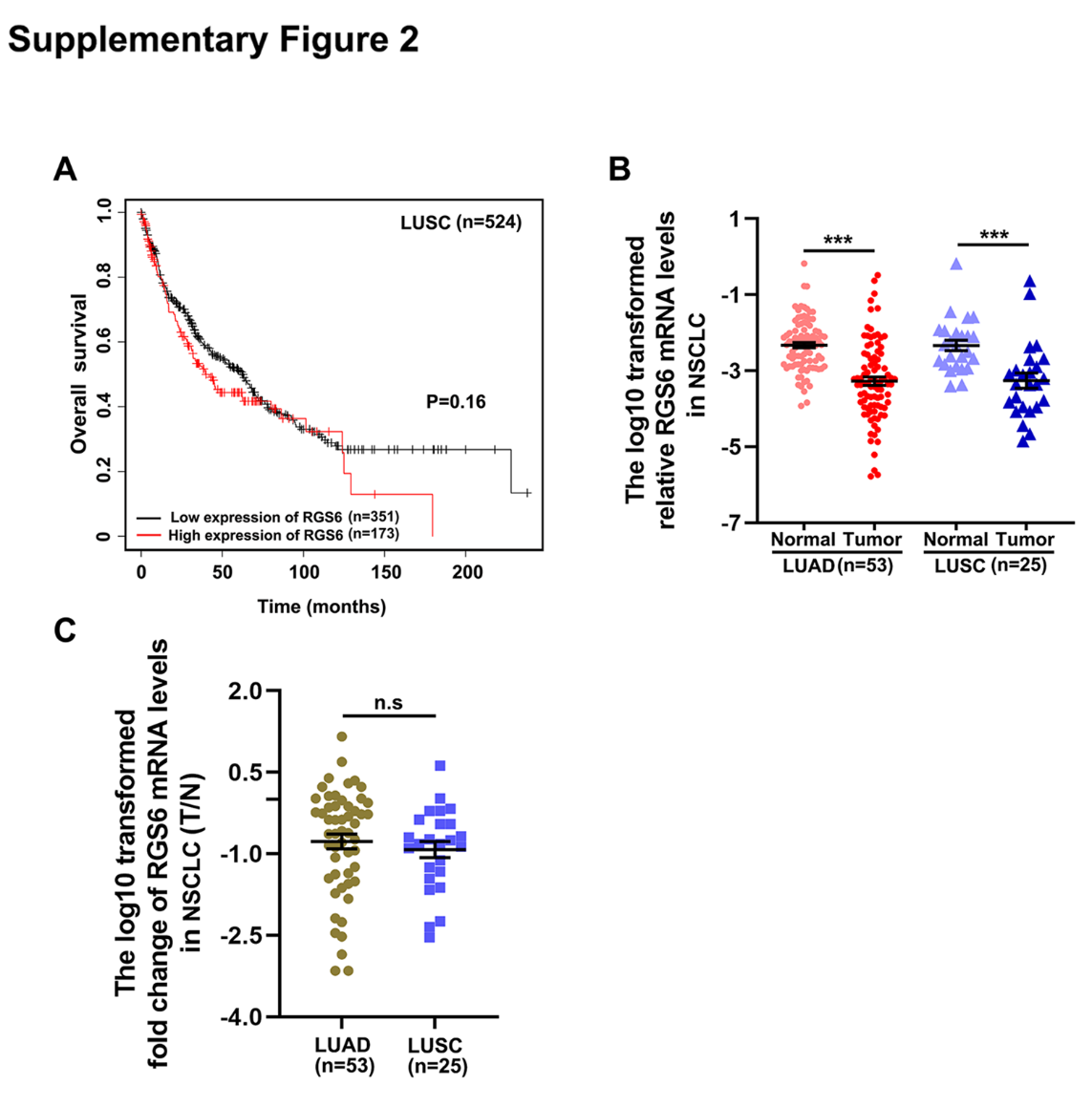


***Supplementary Figure 2. RGS6 expression in NSCLC patients***

(**A**) There is no significant correlation between the level of RGS6 and survival of LUSC patients. Kaplan–Meier plots were generated using Kaplan–Meier Plotter ([*http://www.kmplot.com*](http://www.kmplot.com)). (**B**) qRT-PCR analysis of RGS6 mRNA levels in paired tumor and normal tissues from 78 NSCLC (53 LUAD and 25 LUSC) patients. Mean values are indicted by solid bars, and values are expressed as mean ± SEM. T, NSCLC tissues; N, paired noncancerous lung tissues. (****p*<0.001) (**C**) There is no significant difference between the relative expression (T/N) of RGS6 in LUAD patients and in LUSC patients.


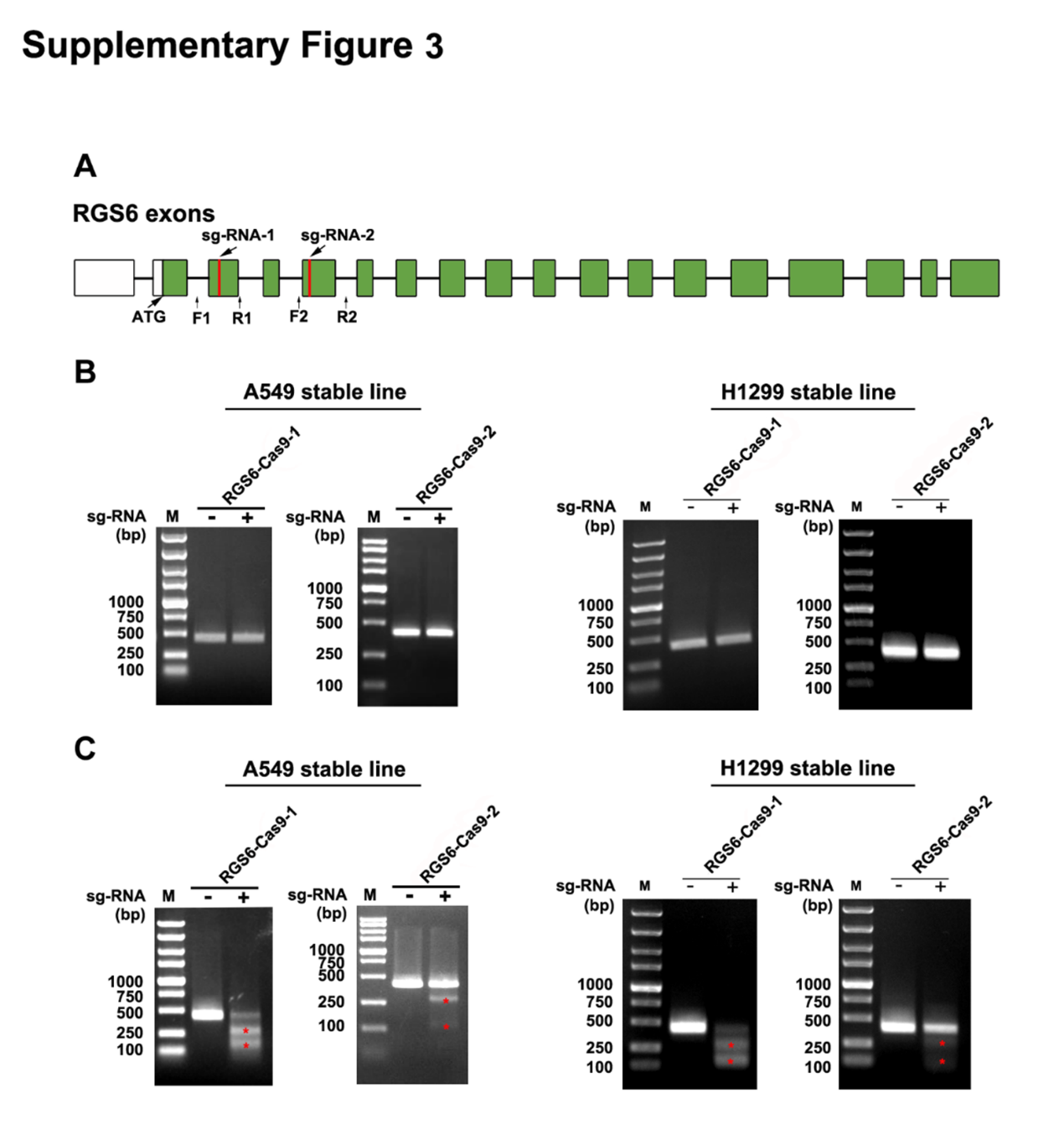


***Supplementary Figure 3. Generation of RGS6 knockdown cells using the CRISPR/Cas9 system***

(**A**) Schematic diagram showing RGS6 gene, exons are shown as filled boxes and noncoding sequences as empty boxes, introns as lines. The red lines indicate the target sites of guide RNA. (**B**) Amplification of the regions bridging the guide RNA target sites by PCR. (**C**) Detection of the target sites in the PCR products with T7 endonuclease I digestion. The fragments of cleavage are indicated by red asterisks.


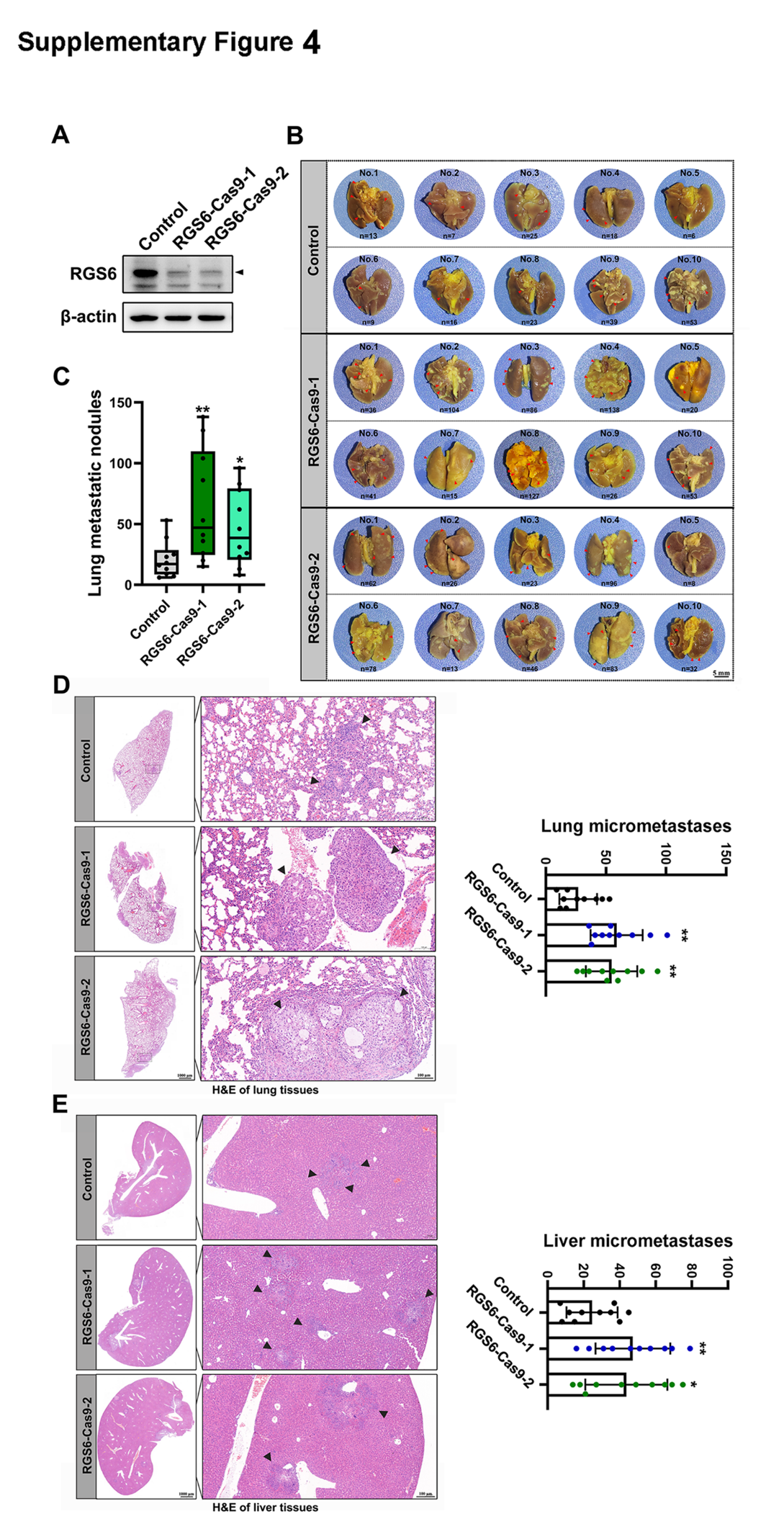


***Supplementary Figure 4. Loss of RGS6 enhances TGF-β-promoted metastasis of NSCLC cells in vivo***

(**A**) RGS6 protein expressed in the A549-Cas9 stable lines were determined by western blotting. (**B**) Representative images of lung tissues from the two RGS6-Cas9 groups and control group, some obvious macroscopically observable metastatic nodules are pointed by red arrow heads. (**C**) Quantification of macroscopic metastatic nodules in lung tissues from each animal of the three groups 7 weeks after inoculation of above cells. (**p* < 0.05; ***p* <0.01) (**D**) Left, representative H&E images of lung tissue sections from animals in the three groups. Micrometastases are pointed by arrow heads. Right, statistical analysis of metastatic nodules in lung tissues from animals in the three groups. (***p* <0.01) (**E**) Left, representative H&E images of liver tissue sections from animals in the three groups. Micrometastases are pointed by arrow heads. Right, statistical analysis of metastatic nodules in liver tissues from animals in the three groups. (**p* < 0.05; ***p* <0.01)


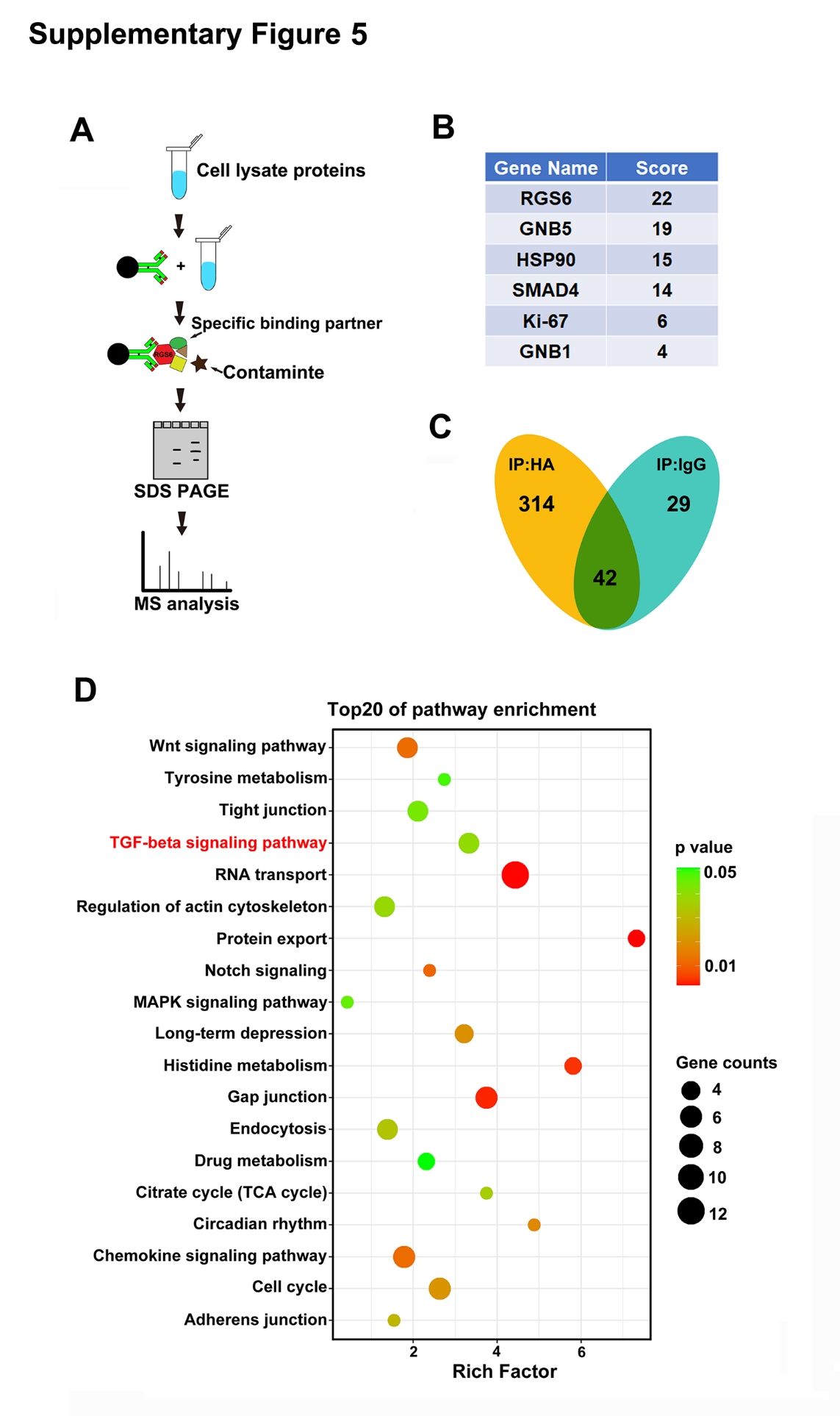


***Supplementary Figure 5. Mass spectrometry analysis identifying RGS6-associated proteins***

(**A**) Schematic diagram showing process of mass spectrometry analysis to identify RGS6-associated proteins. (**B**, **C**) List of proteins identified in RGS6 immunoprecipitates by mass spectroscopy analysis. (**D**) KEGG pathway enrichment analysis of data obtained from RGS6 immunoprecipitation.

、
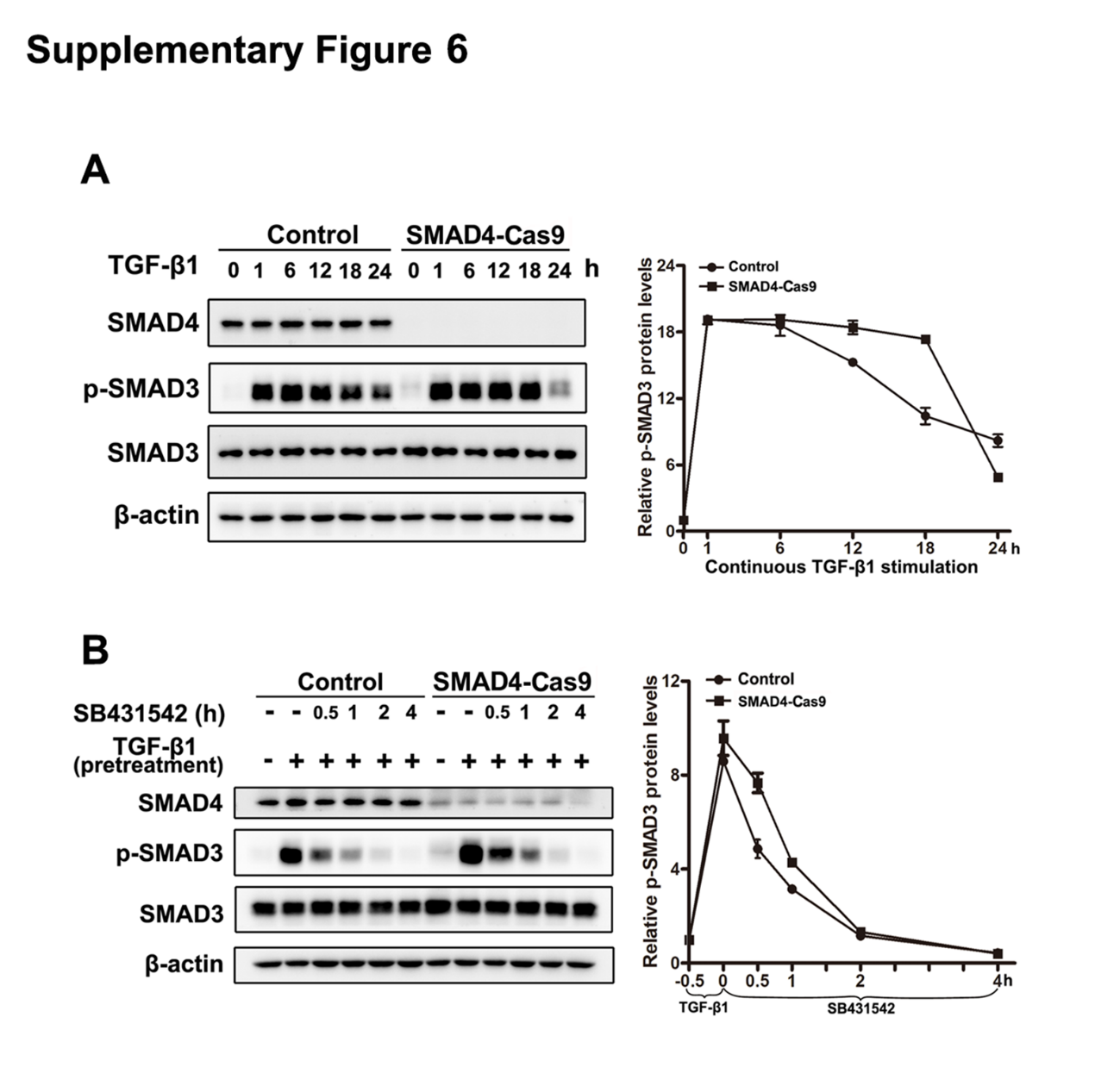


***Supplementary Figure 6. Kinetics of TGF-β-induced SMAD3 phosphory-lation in SMAD4-KO NSCLC cells***

**(**A) SMAD4-KO (SMAD4-Cas9) A549 cells and control A549 cells were treated with TGF-β (5 ng/ml) for the indicated time periods, and subjected to western blotting for detection of indicated proteins. Left: a representative western blotting. Right, quantification of intensity of p-SMAD3 relative to total SMAD3 in three independent experiments using Image J software. (**B**) SMAD4-Cas9 cells and control A549 were treated with TGF-β (5ng/ml) for 30 min, followed by TGF-β washout and simultaneous addition of 5 mM SB431542. After treatment of SB431542 for indicated time periods, cell lysates were collected and blotted for indicated proteins. Left: a representative western blotting. Right, quantification of intensity of p-SMAD3 relative to total SMAD3 in three independent experiments using Image J software.


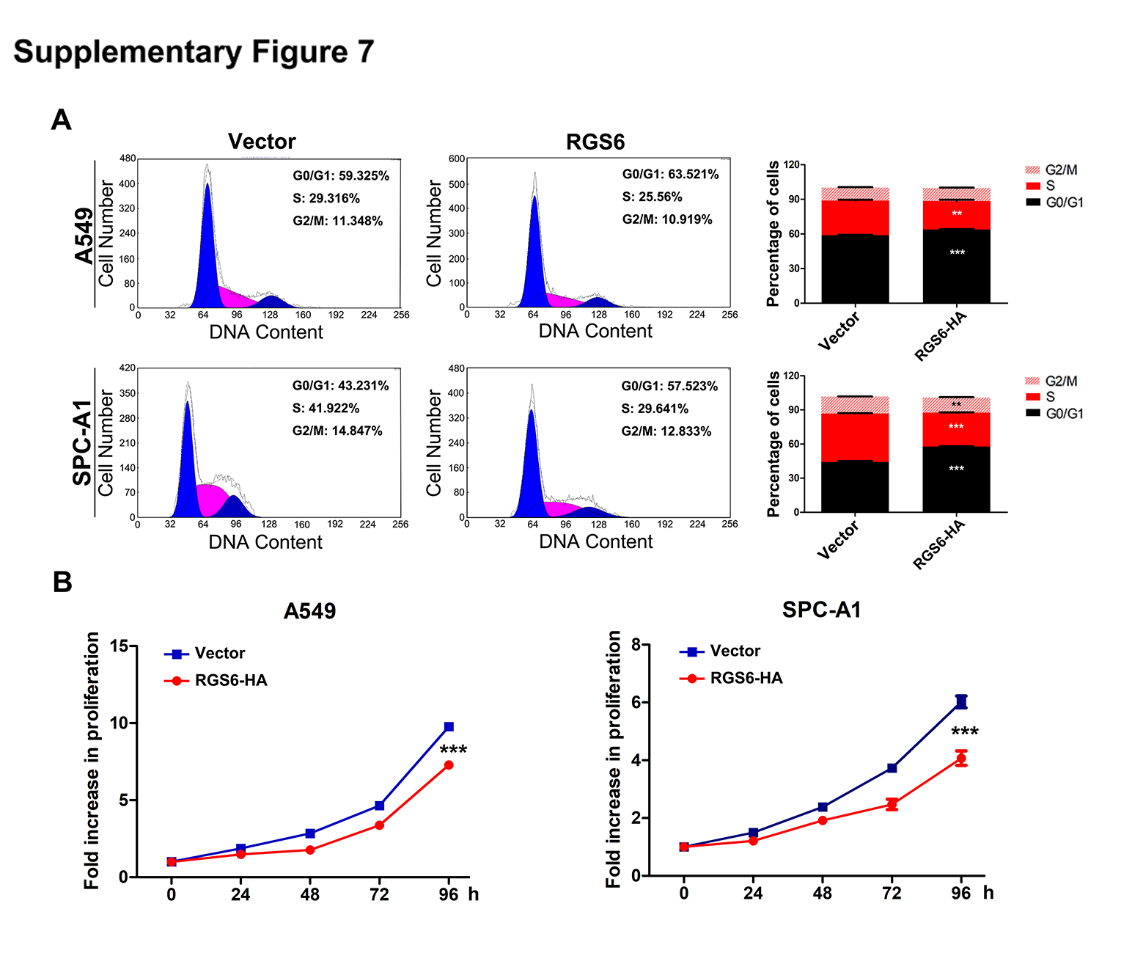


***Supplementary Figure 7. An inhibitory effect of RGS6 on cell growth of NSCLC cells***

(A) Cell cycle progression of RGS6-overexpressing and controlled NSCLC cells were examined using flow cytometry. (**p<0.01, ***p<0.001) (B) Cell proliferation of control and RGS6-overexpressing NSCLC cells were measured using CCK-8 assay. (***p<0.001)
