# Supplementary material for: RGS6 suppresses TGF-β-induced epithelial–mesenchymal transition in non-small cell lung cancers via a novel mechanism dependent on its interaction with SMAD4
Source: Cell Death Dis. 2022 Jul 28;13(7):656. doi: 10.1038/s41419-022-05093-0 (PMC9334288; doi:10.1038/s41419-022-05093-0)
Supplement: Supplementary file 3 — Supplementary Materials and Methods [file 41419_2022_5093_MOESM3_ESM.docx]

**Supplementary Materials and Methods**

**Cell culture and transfection**

The human NSCLC cells A549 and H1299 were obtained from the Cell Bank of Chinese Academy of Sciences (Shanghai, China). Cell lines have been authenticated by sequencing and were tested monthly for mycoplasma contamination. Cells were cultured in RPMI 1640 medium (HyClone, South Logan, UT, USA) containing 10% fetal bovine serum (FBS, Gibco, Carlsbad, CA, USA) and 1% penicillin/streptomycin (HyClone) at 37°C in a humidified atmosphere containing 5% CO_2_. Cells were transiently transfected with Lipofectamine 3000 Reagent (Invitrogen, Waltham, MA, USA), using the manufacturer's standard protocol.

**Tissue samples**

Paired fresh frozen human lung cancer tissues and adjacent noncancerous lung tissues were collected after informed consent from patients in the First Affiliated Hospital of Soochow University. All patients had been diagnosed with lung cancer followed by histological and pathological characteristics according to the Revised International System for Staging Lung Cancer, and patients received neither chemotherapy nor radiotherapy before tissue sampling. Clinical characteristics of the patients are detailed in Supplementary Table S1, metastatic tissues (n=48) were form patients with local lymph node metastasis (T_1–4_N_1–2_M_0_) or distant organ metastasis (T_1–4_N_any_M_1_), and non-metastatic tissues (n=44) were from patients without any metastasis (T_1–4_N_0_M_0_). This study was approved by the Ethics Committee of Soochow University.

**RNA extraction, cDNA synthesis, and quantitative real-time PCR (qRT-PCR)**

Total RNA was isolated from cultured cells and tissues using TRIzol (Thermo Fisher Scientific, Carlsbad, CA, USA) in accordance with the manufacturer's instructions. cDNA synthesis using a M-MLV reverse transcriptase kit (Vazyme, Nanjing, China), followed by qRT–PCR analysis (SYBR Green; Vazyme) according to the manufacturer’s instructions. Primers are listed in (Supplementary Table S6). GAPDH was used as the endogenous control for RGS6, Snail, PAI-1 and SMAD2/3/4 mRNA. Relative expression of each RNA was determined using the ΔΔCt method. Each qRT-PCR analysis was done in triplicates.

**Western blotting assay and nuclear fractionation**

Western blotting was carried out as described previously[29]. Antibodies used in the western blot analysis were as follows: anti-RGS6 (Abcam, London, UK; 1:1,000; #ab128943); Anti-E-cadherin, anti-N-cadherin and anti-Vimentin (BD Biosciences, CA, USA; 1:3,000, #610181, #610920, #550513); anti-Snail, anti-SMAD2, anti-p-SMAD2(ser465/ser467), anti-SMAD3, anti-p-SMAD3(ser423/ser425), anti-SMAD4, anti-HA and anti-Flag (Cell Signaling Technology, MA, USA; 1:3000; #3895, #5339, #18338, #9523, #9520, #46535, #3724, #14793 ); Anti-β-actin and anti-mouse or-rabbit secondary antibodies (Santa Cruz Biotechnology, CA, USA; 1:3,000; #sc-47778, #sc-2005, #sc-2004). The Nuclear and Cytoplasmic Protein Extraction Kit (Sangon Biotech; Shanghai; China; #c510001) was applied to isolate cytosolic and nuclear fractions in A549 cells. After separation, samples for analysis by SDS-PAGE and immunoblotting.

**Construction of HA-tagged RGS6 and Flag-tagged SMAD4 expression vectors**

GFP-tagged RGS6 (full length and deletion mutants) expressing vectors were kindly provided by Prof. Fisher Rory. For construction of the plasmids expressing HA-tagged RGS6 and Flag-tagged SMAD4, and its truncation mutants, RGS6 and SMAD4 genes were amplified with the corresponding primers (supplementary Table S6), then subcloned into the pcDNA3.1-HA/pcDNA3.1-Flag plasmid which were kindly provided by Dr. Yuanyuan Zeng. The sequences of all constructs were confirmed by direct sequencing.

**Generation of stable cell lines overexpressing RGS6**

To generate A549 and H1299 cell lines stably overexpressing RGS6, the full-length human RGS6 gene was subcloned into a pLenti-GIII-CMV-GFP-2A-Puro overexpression vector (Applied Biological Materials Inc., Zhenjiang, China) with restriction endonucleases *Nhe*I and *Xba*I. The RGS6 expression construct was co-transfected with packaging plasmids into HEK293T cells using lipo3000 (Invitrogen). The above cells were cultured for 48 hrs. After incubation, the supernatants were collected and used to infect A549 and H1299 cells. Forty-eight hours after infection, stable cells were selected with 2.0 μg/ml puromycin (Applied Biological Materials).

**Production of RGS6 Knockout cells by the CRISPR/Cas9 system**

Two single guide RNAs (sgRNA) sequences (Supplementary Table S5) targeting RGS6 exon 3 and 5 were designed using the online CRISPR design tool (*<https://zlab.bio/guide-design-resources>*). Cloning of sgRNAs into Lenti-sgRNA-EGFP vector (Genechem Co., Shanghai, China) with restriction endonucleases *Bbs*I, and then co-infected with Lenti-Cas9-puro in NSCLC cells. Single-cell clones were selected and PCR products of target sites were detected with T7 endonuclease I assay (New England Biolabs; MA, USA) for confirmation of mutations. Lastly, the obtained PCR products were subcloned into pMD^TM^ 19-T vector (Takara Biotechnology Co. Ltd; Dalian, China) and were confirmed by direct sequencing.

**Co-immunoprecipitation (co-IP)**

Briefly, whole cell lysates were prepared with lysis buffer (150 mM NaCl, 10 mM HEPES, pH 7.4 and 1% NP-40) containing protease inhibitors (Bimake; Houston, TX, USA) and phosphatase inhibitors (Bimake) for 30 mins on ice, then sheared by sonication (3×10 s) in tubes. Total protein was incubated with an anti-Flag or anti-HA Beads (Bimake) overnight at 4℃ according to the manufacturer’s instructions. Lastly, immunoprecipitated samples were subjected to immunoblotting.

**Immunohistochemistry (IHC)**

Immunohistochemistry was performed on the sections (5 μm thickness) from the lung TMA. Antigen retrieval was performed with citrate buffer (pH 6.0), using a pressure cooker set 120°C for 15 mins. The sections were incubated with primary antibody to RGS6 (dilution 1:200) overnight at 4°C. Subsequently, immunodetection was made using DAB kit (Dako, USA) according to the manufacturer’s instructions. All stains were evaluated by a single pathologist (HM) blinded to sample identify.

**Migration and invasion assays**

The capacity of cells to migrate or invade were evaluated through Transwell assay. In briefly, 5×10^4^ cells in 1% FBS medium were inoculated onto the upper chamber of transwell insert without (for migration) or with precoated Matrigel matrix (Corning, NY, USA) (for invasion), and 800 μl of 20% FBS medium was added to the lower chamber. 6 hrs later, TGF-β was added to the chamber and were incubated for 24 hrs at 37°C to allow the cells to migrate to the lower well. The cells that had migrated through the membrane were fixed in methanol, stained with 1% crystal violet. Finally, the cells were counted in at least three random fields under a light microscope

**Wound-healing assay**

Briefly, cells were seeded onto 6-well cell culture plates at 37°C in 5% CO2 and incubated for 24 hrs. The cell monolayers were mechanically scratched using a sterile 10-μl pipette tip to create a linear wound. Wound healing within the gap was observed in specified times at the same position using an inverted microscope. And rate of wound healing was then evaluated by estimating the area of healed wound until the wound was completely healed. The gap was analyzed using ImageJ software and each experiment was repeated in triplicates.

**Immunofluorescence staining**

In briefly, cells were transfected with the designated expression plasmids for 48 hrs. The cells were then fixed with 4% paraformaldehyde and permeabilized with 0.1% Triton *X*-100 in PBST, incubated overnight with primary antibodies at 4°C and subsequently incubated for 2 hrs with fluorescently-labeled secondary antibodies (Beyotime, Jiangsu, China). Finally, cellular nuclei were stained with DAPI.

**Dual-luciferase reporter assay**

Briefly, the luciferase reporter vector containing *PAI-1* promoter and TPA responsive elements sequence was transfected into A549 cells for 48 hrs. Subsequent luciferase reporter assay was performed using the dual-luciferase reporter gene assay kit (Yeasen Biotech, Shanghai, China) according to the manufacturer’s protocol. The Luciferase reporter assay was repeated at least three times in parallel for statistical analysis.

**Tumor xenografts and metastasis models**

BALB/c nude mice (4-6 weeks old, female) were purchased from the Experimental Animal Center of Soochow University and maintained under specific pathogen-free conditions. Experimental metastasis model was generated by tail-vein injection of RGS6-HA stable or control A 549 cells (3 × 106 cells/mice). To ensure statistical significance, each group contained 10 mice. Randomization was not applied in this study. TGF-β (4 μg/kg) was intraperitoneally injected every 5th day post cell inoculation. Eight weeks later, the mice were euthanized and their lung, liver tissues were taken out and fixated in universal tissue fixative. Macroscopically observable metastatic nodules on surface of each tissue were counted and subjected to sectioning and histologically analysis with H&E staining. No blinding was done in this study. Animal studies were approved and supervised by the Animal Ethics Committee of Soochow University.

**Statistical analysis**

Results were presented as mean ± SD. Difference between two groups was assessed using paired t test (2-tailed) and Pearson’s correlation coefficient test was used to evaluate the association between two groups of patient samples. Differences with *p* < 0.05 were considered significant. All statistical analyses were conducted using GraphPad Prism 7 software (GraphPad, San Diego, CA, USA).
