# Supplementary material for: RGS6 suppresses TGF-β-induced epithelial–mesenchymal transition in non-small cell lung cancers via a novel mechanism dependent on its interaction with SMAD4
Source: Cell Death Dis. 2022 Jul 28;13(7):656. doi: 10.1038/s41419-022-05093-0 (PMC9334288; doi:10.1038/s41419-022-05093-0)
Supplement: Supplementary file 4 — Supplmentary Table S1 [file 41419_2022_5093_MOESM4_ESM.docx]

**Table S1. Demographic characteristics of 92 lung cancer patients and relative expression of RGS6 mRNA in 92 paired lung cancer tissues**

| **Case** | **Age (years)** | **Sex** | **Histology ^*^** | **TNM** | **Stage** | **RGS6(T/N)** |
| --- | --- | --- | --- | --- | --- | --- |
| 1  2  3  4  5  6  7  8  9  10  11  12  13  14  15  16  17  18  19  20  21  22  23  24  25  26  27  28  29  30  31  32  33  34  35  36  37  38  39  40  41  42  43  44  45  46  47  48  49  50  51  52  53  54  55  56  57  58  59  60  61  62  63  64  65  66  67  68  69  70  71  72  73  74  75  76  77  78  79  80  81  82  83  84  85  86  87  88  89  90  91  92 | 70  54  65  73  57  65  64  60  64  63  62  67  65  35  77  72  73  69  62  72  58  72  87  85  64  55  62  57  63  54  62  75  32  71  66  60  62  61  59  49  59  67  40  52  78  52  74  65  63  71  73  67  48  65  64  66  65  55  58  67  70  72  71  57  64  68  56  65  36  69  61  42  61  73  71  69  77  68  68  58  73  71  47  60  57  39  54  53  60  60  49  47 | male  female  female  male  male  male  male  male  male  female  male  male  male  male  male  male  male  male  male  female  male  male  male  male  male  male  male  male  female  female  male  male  male  female  male  male  male  male  male  male  female  male  female  female  female  female  male  female  female  female  male  male  female  male  male  male  male  female  male  female  male  female  female  female  female  male  female  male  female  female  male  female  female  male  male  male  male  male  male  male  male  female  male  female  female  female  male  female  male  male  male  male | LUSC  LUAD  LUAD  LUAD  LUAD  LUAD  LUSC  LUSC  LUSC  Other  LUAD  LUAD  LUAD  LUAD  LUAD  LUSC  LUSC  LUAD  Other  LUAD  LUAD  LUSC  LUAD  Other  LUSC  LUSC  LUAD  LUAD  Other  LUAD  LUSC  Other  LUAD  LUAD  LUAD  LUAD  LUAD  Other  LUAD  LUAD  LUAD  LUAD  LUAD  LUAD  LUAD  LUAD  LUSC  LUAD  LUAD  LUAD  Other  LUSC  LUSC  LUAD  LUSC  LUAD  Other  LUAD  LUSC  LUAD  LUSC  LUAD  LUAD  LUAD  LUAD  LUAD  LUAD  LUSC  Other  LUAD  LUSC  LUAD  Other  LUSC  LUSC  LUAD  Other  LUSC  LUAD  Other  LUSC  LUAD  Other  LUAD  LUAD  LUAD  LUSC  LUAD  LUAD  LUSC  LUSC  Other | T2bN1M0  T1bN0M0  T3N2M0  T1bN0M0  T2aN1M0  T2aN1M0  T1cN0M0  T2aN1M0  T2aN0M0  T1cN0M1a  T1bN0M0  T4N0M0  T1cN0M0  T2aN2M0  T1bN2M0  T2aN2M0  T1bN0M0  T2aN2M0  T2bN2M0  T2aN0M0  T3N0M0  T2aN2M0  T2bN0M0  T3N0M0  T2bN2M0  T1cN2M0  T1bN1M0  T2aN1M0  T1cN0M1a  T1bN0M0  T2aN2M0  T2bN2M0  T1bN1M1c  T1bN0M0  T2aN0M0  T2aN0M0  T2aN2M0  T2bN2M0  T4N2M0  T2bN1M0  T2bN2M0  T2aN0M0  T1cN1M0  T2aN0M0  T4N1M0  T2aN0M0  T2bN2M0  T1bN0M0  T1cN1M0  T2aN0M0  T1cN0M0  T2bN0M0  T2aN0M0  T1cN0M0  T1bN0M0  T1cN0M1  T2aN2M0  T2aN2M0  T2aN1M0  T3N0M0  T2aN2M0  T2aN0M0  T2aN0M0  T2aN0M0  T4N2M1b  T1bN1M0  T2bN2M0  T2bN0M0  T1bN1M0  T2aN0M0  T1bN0M0  T2aN0M0  T2aN1M0  T3N2M0  T2bN0M0  T2aN0M1a  T3N0M0  T4N2M0  T2aN0M0  T3N0M0  T3N0M0  T2aN0M0  T3N2M0  T1bN2M0  T2aN2M0  T1aN0M0  T4N1M0  T2aN0M0  T4N0M1a  T4N1M0  T3N1M0  T4N0M1b | Ⅱb  Ia2  Ⅲb  Ia2  Ⅱb  Ⅱb  Ia3  Ⅱb  Ⅰb  Ⅳa  Ia2  Ⅲa  Ⅰa3  Ⅲa  Ⅲa  Ⅲa  Ⅰa2  Ⅲa  Ⅲa  Ⅰb  Ⅱb  Ⅲa  Ⅱa  Ⅱb  Ⅲa  Ⅲa  Ⅱb  Ⅱb  Ⅳa  Ⅰa2  Ⅲa  Ⅲa  Ⅳb  Ⅰa2  Ⅰb  Ⅰb  Ⅲa  Ⅲa  Ⅲb  Ⅱb  Ⅲa  Ⅰb  Ⅱb  Ⅰb  Ⅲa  Ⅰb  Ⅲa  Ⅰa2  Ⅱb  Ⅰb  Ⅰa3  Ⅱb  Ⅰb  Ⅰa3  Ⅰa2  Ⅳa  Ⅲa  Ⅲa  Ⅱb  Ⅱb  Ⅲa  Ⅰb  Ⅰb  Ⅰb  Ⅳa  Ⅱb  Ⅲa  Ⅱa  Ⅱb  Ⅰb  Ⅰa  Ⅰb  Ⅱa  Ⅲb  Ⅱa  Ⅳa  Ⅱb  Ⅲb  Ⅰb  Ⅱb  Ⅱb  Ⅰb  Ⅲb  Ⅲa  Ⅲa  Ⅰa1  Ⅲa  Ⅰb  Ⅳa  Ⅲa  Ⅲa  Ⅳa | 0.0057  0.8679  0.9594  0.4224  0.3590  0.4245  0.0562  0.1530  0.3508  0.0165  0.5335  1.7093  1.9867  0.0439  1.0421  0.3539  0.1768  0.1329  0.0053  0.0007  0.7524  4.2096  14.3285  0.0099  0.4220  0.1359  1.6994  0.0014  0.0022  1.1820  0.1260  0.0375  0.2333  0.0574  0.0280  0.6158  4.9092  0.0461  0.0352  0.2421  0.0859  0.5555  0.0237  0.5342  0.1839  0.7469  0.0470  0.5804  0.0188  0.0007  0.0191  0.2008  0.2099  0.0065  0.0754  0.0420  0.1217  0.0148  0.6171  0.0055  0.0045  0.0030  0.1152  2.2370  0.7122  0.1429  0.1606  1.0386  0.0502  1.1519  0.6693  2.4658  0.3525  0.0217  0.6135  1.0455  0.0659  0.0350  0.2602  0.0011  0.1843  0.4886  0.0008  0.0035  0.2316  0.0311  0.1520  0.1062  0.4216  0.0238  0.0029  1.8860 |

^*^ LUAD, Lung adenocarcinoma; LUSC, Lung squamous cell carcinoma; Other, large cell carcinoma, etc.

Metastatic tissues (n=48) were form patients with local lymph node metastasis (T_1–4_N_1–2_M_0_) or distant organ metastasis (T_1–4_N_any_M_1_), and non-metastatic tissues (n=44) were from patients without any metastasis (T_1–4_N_0_M_0_).

T, tumor tissues; N, paired noncancerous lung tissues. Ratio values of T/N > 1.0, T/N < 1.0 and T/N ≈ 1.0 represent increased, reduced and preserved expression, respectively.
