# Supplementary material for: RGS6 suppresses TGF-β-induced epithelial–mesenchymal transition in non-small cell lung cancers via a novel mechanism dependent on its interaction with SMAD4
Source: Cell Death Dis. 2022 Jul 28;13(7):656. doi: 10.1038/s41419-022-05093-0 (PMC9334288; doi:10.1038/s41419-022-05093-0)
Supplement: Supplementary file 5 — Supplmentary Table S2 [file 41419_2022_5093_MOESM5_ESM.docx]

**Table S2. Clinical characteristics of 92 lung cancer patients and levels of RGS6 expression in 92 paired lung cancer tissues**

| **Characteristics** | **n=92** | **RGS6 mRNA expression** | | **P value** |
| --- | --- | --- | --- | --- |
|  |  | **low(n=46)** | **high(n=46)** |  |
| **Age (years)**  **≤60**  **<60**  **Gender**  **male**  **female** | **63 (68.5%)**  **29 (31.5%)**  **60 (65.2%)**  **32 (34.8%)** | **32**  **15**  **31**  **17** | **31**  **14**  **29**  **15** | **0.934**  **0.894** |
| **Histological features**  **AD**  **SCC** | **53 (57.6%)**  **25 (27.2%)** | **23**  **13** | **30**  **12** | **0.477** |
| **Degree of differentiation**  **low**  **high** | **52 (56.5%)**  **40 (43.5%)** | **27**  **19** | **25**  **21** | **0.674** |
|  |  |  |  |  |
| **Clinical stage**  **Ⅰ + Ⅱ**  **Ⅲ +Ⅳ** | **73 (79.3%)**  **19 (20.7%)** | **35**  **11** | **38**  **8** | **0.439** |
| **Distant metastasis**  **No**  **Yes** | **44 (47.8%)**  **48 (52.2%)** | **13**  **33** | **31**  **15** | **＜0.001** |
|  |  |  |  |  |

**AD,** **Adenocarcinoma; SCC, Squamous cell carcinoma.**
