# Supplementary material for: RGS6 suppresses TGF-β-induced epithelial–mesenchymal transition in non-small cell lung cancers via a novel mechanism dependent on its interaction with SMAD4
Source: Cell Death Dis. 2022 Jul 28;13(7):656. doi: 10.1038/s41419-022-05093-0 (PMC9334288; doi:10.1038/s41419-022-05093-0)
Supplement: Supplementary file 6 — Supplmentary Table S3 [file 41419_2022_5093_MOESM6_ESM.docx]

**Table S3. Demographic and clinical characteristics of 75 lung adenocarcinoma patients and the cumulative score of RGS6 IHC staining in paired tumor tissues**

| **Case** | **Age**  **(years)** | **Sex** | **Tissue type** | **Stage** | **Staining intensity** | **Positive** | **Cumulative score** |
| --- | --- | --- | --- | --- | --- | --- | --- |
| **1** | **53** | **female** | **tumor** | **II** | **1+** | **100%** | **4** |
|  |  |  | **normal** |  | **2+** | **100%** | **8** |
| **2** | **67** | **male** | **tumor** | **II-III** | **1+** | **100%** | **4** |
|  |  |  | **normal** |  | **2+** | **100%** | **8** |
| **3** | **73** | **male** | **tumor** | **II** | **1+** | **100%** | **4** |
|  |  |  | **normal** |  | **2+** | **100%** | **8** |
| **4** | **70** | **male** | **tumor** | **I** | **1+** | **100%** | **4** |
|  |  |  | **normal** |  | **2+** | **100%** | **8** |
| **5** | **69** | **female** | **tumor** | **II** | **1+** | **100%** | **4** |
|  |  |  | **normal** |  | **1+** | **100%** | **4** |
| **6** | **73** | **male** | **tumor** | **II** | **1+** | **100%** | **4** |
|  |  |  | **normal** |  | **2+** | **100%** | **8** |
| **7** | **61** | **female** | **tumor** | **II** | **1+** | **100%** | **4** |
|  |  |  | **normal** |  | **1-2+** | **100%** | **6** |
| **8** | **70** | **female** | **tumor** | **II-III** | **1+** | **100%** | **4** |
|  |  |  | **normal** |  | **1+** | **100%** | **4** |
| **9** | **54** | **male** | **tumor** | **II-III** | **1-2+** | **100%** | **6** |
|  |  |  | **normal** |  | **2+** | **100%** | **8** |
| **10** | **68** | **male** | **tumor** | **II-III** | **1+** | **100%** | **4** |
|  |  |  | **normal** |  | **2+** | **100%** | **8** |
| **11** | **40** | **female** | **tumor** | **II** | **2+** | **100%** | **8** |
|  |  |  | **normal** |  | **1+** | **100%** | **4** |
| **12** | **51** | **male** | **tumor** | **II** | **1+** | **＜10%** | **1** |
|  |  |  | **normal** |  | **1+** | **＜10%** | **1** |
| **13** | **59** | **male** | **tumor** | **II-III** | **1+** | **100%** | **4** |
|  |  |  | **normal** |  | **3+** | **100%** | **12** |
| **14** | **68** | **female** | **tumor** | **II** | **1+** | **100%** | **4** |
|  |  |  | **normal** |  | **2+** | **100%** | **8** |
| **15** | **52** | **male** | **tumor** | **III** | **1+** | **＜1%** | **1** |
|  |  |  | **normal** |  | **1+** | **100%** | **4** |
| **16** | **63** | **male** | **tumor** | **II** | **1+** | **100%** | **4** |
|  |  |  | **normal** |  | **2+** | **100%** | **8** |
| **17** | **65** | **male** | **tumor** | **III** | **1+** | **60%** | **3** |
|  |  |  | **normal** |  | **1+** | **100%** | **4** |
| **18** | **54** | **male** | **tumor** | **II- III** | **1+** | **100%** | **4** |
|  |  |  | **normal** |  | **1+** | **100%** | **4** |
| **19** | **72** | **female** | **tumor** | **II** | **1-2+** | **100%** | **6** |
|  |  |  | **normal** |  | **2+** | **100%** | **8** |
| **20** | **64** | **female** | **tumor** | **II** | **1+** | **100%** | **4** |
|  |  |  | **normal** |  | **1+** | **100%** | **4** |
| **21** | **72** | **male** | **tumor** | **II** | **1-2+** | **100%** | **6** |
|  |  |  | **normal** |  | **3+** | **100%** | **12** |
| **22** | **63** | **female** | **tumor** | **II** | **2+** | **100%** | **8** |
|  |  |  | **normal** |  | **1+** | **100%** | **4** |
| **23** | **74** | **male** | **tumor** | **II** | **1+** | **100%** | **4** |
|  |  |  | **normal** |  | **2+** | **100%** | **8** |
| **24** | **38** | **female** | **tumor** | **II** | **1+** | **100%** | **4** |
|  |  |  | **normal** |  | **1+** | **100%** | **4** |
| **25** | **49** | **female** | **tumor** | **II-III** | **1-2+** | **100%** | **6** |
|  |  |  | **normal** |  | **1+** | **100%** | **4** |
| **26** | **60** | **female** | **tumor** | **II** | **1+** | **＜1%** | **0** |
|  |  |  | **normal** |  | **1+** | **10%** | **1** |
| **27** | **35** | **male** | **tumor** | **II** | **2+** | **100%** | **8** |
|  |  |  | **normal** |  | **2+** | **100%** | **8** |
| **28** | **63** | **male** | **tumor** | **II-III** | **1+** | **100%** | **4** |
|  |  |  | **normal** |  | **2+** | **100%** | **8** |
| **29** | **61** | **female** | **tumor** | **II-III** | **1+** | **100%** | **4** |
|  |  |  | **normal** |  | **2+** | **100%** | **8** |
| **30** | **64** | **- -** | **tumor** | **II** | **2+** | **100%** | **8** |
|  |  |  | **normal** |  | **1+** | **100%** | **4** |
| **31** | **45** | **female** | **tumor** | **II** | **2+** | **100%** | **8** |
|  |  |  | **normal** |  | **1+** | **100%** | **4** |
| **32** | **64** | **female** | **tumor** | **II** | **1+** | **100%** | **4** |
|  |  |  | **normal** |  | **2+** | **100%** | **8** |
| **33** | **45** | **female** | **tumor** | **II** | **1+** | **＜1%** | **0** |
|  |  |  | **normal** |  | **1+** | **100%** | **4** |
| **34** | **45** | **female** | **tumor** | **II-III** | **2+** | **100%** | **8** |
|  |  |  | **normal** |  | **1+** | **100%** | **4** |
| **35** | **63** | **female** | **tumor** | **II-III** | **1+** | **100%** | **4** |
|  |  |  | **normal** |  | **1+** | **100%** | **4** |
| **36** | **49** | **male** | **tumor** | **II** | **2+** | **100%** | **8** |
|  |  |  | **normal** |  | **2+** | **100%** | **8** |
| **37** | **77** | **male** | **tumor** | **III** | **1+** | **100%** | **4** |
|  |  |  | **normal** |  | **2+** | **100%** | **8** |
| **38** | **60** | **male** | **tumor** | **II-III** | **1+** | **100%** | **4** |
|  |  |  | **normal** |  | **2+** | **100%** | **8** |
| **39** | **47** | **male** | **tumor** | **III** | **1+** | **100%** | **4** |
|  |  |  | **normal** |  | **1+** | **＜1%** | **0** |
| **40** | **52** | **female** | **tumor** | **II** | **1+** | **100%** | **4** |
|  |  |  | **normal** |  | **2+** | **100%** | **8** |
| **41** | **56** | **male** | **tumor** | **III** | **1+** | **＜1%** | **0** |
|  |  |  | **normal** |  | **2+** | **100%** | **8** |
| **42** | **60** | **male** | **tumor** | **II** | **1+** | **＜1%** | **0** |
|  |  |  | **normal** |  | **1+** | **100%** | **4** |
| **43** | **66** | **female** | **tumor** | **II** | **1+** | **5%** | **1** |
|  |  |  | **normal** |  | **2+** | **5%** | **1** |
| **44** | **63** | **female** | **tumor** | **II** | **1+** | **＜1%** | **0** |
|  |  |  | **normal** |  | **1+** | **＜1%** | **0** |
| **45** | **76** | **female** | **tumor** | **II** | **1+** | **100%** | **4** |
|  |  |  | **normal** |  | **1+** | **100%** | **4** |
| **46** | **64** | **female** | **tumor** | **II** | **1+** | **100%** | **4** |
|  |  |  | **normal** |  | **1+** | **100%** | **4** |
| **47** | **59** | **female** | **tumor** | **II** | **1+** | **100%** | **4** |
|  |  |  | **normal** |  | **3+** | **100%** | **12** |
| **48** | **65** | **female** | **tumor** | **II** | **1+** | **100%** | **4** |
|  |  |  | **normal** |  | **2+** | **100%** | **6** |
| **49** | **58** | **female** | **tumor** | **II** | **1+** | **＜1%** | **0** |
|  |  |  | **normal** |  | **2+** | **5%** | **2** |
| **50** | **59** | **female** | **tumor** | **II** | **1+** | **＜1%** | **0** |
|  |  |  | **normal** |  | **1+** | **＜1%** | **0** |
| **51** | **65** | **female** | **tumor** | **II** | **1+** | **＜1%** | **0** |
|  |  |  | **normal** |  | **2+** | **100%** | **8** |
| **52** | **48** | **male** | **tumor** | **II** | **1+** | **＜1%** | **0** |
|  |  |  | **normal** |  | **1+** | **100%** | **4** |
| **53** | **56** | **male** | **tumor** | **II** | **1+** | **100%** | **4** |
|  |  |  | **normal** |  | **1+** | **100%** | **4** |
| **54** | **62** | **female** | **tumor** | **II** | **1+** | **100%** | **4** |
|  |  |  | **normal** |  | **2+** | **100%** | **8** |
| **55** | **60** | **male** | **tumor** | **II** | **1+** | **100%** | **4** |
|  |  |  | **normal** |  | **1+** | **100%** | **4** |
| **56** | **65** | **male** | **tumor** | **II-III** | **1+** | **100%** | **4** |
|  |  |  | **normal** |  | **2+** | **100%** | **8** |
| **57** | **50** | **male** | **tumor** | **II-III** | **2+** | **100%** | **8** |
|  |  |  | **normal** |  | **2+** | **100%** | **8** |
| **58** | **68** | **male** | **tumor** | **II** | **1-2+** | **100%** | **6** |
|  |  |  | **normal** |  | **1+** | **100%** | **4** |
| **59** | **58** | **male** | **tumor** | **II** | **1+** | **100%** | **4** |
|  |  |  | **normal** |  | **1-2+** | **100%** | **6** |
| **60** | **61** | **male** | **tumor** | **II** | **1+** | **＜1%** | **0** |
|  |  |  | **normal** |  | **2+** | **100%** | **8** |
| **61** | **42** | **male** | **tumor** | **II-III** | **2+** | **100%** | **8** |
|  |  |  | **normal** |  | **1+** | **100%** | **4** |
| **62** | **64** | **male** | **tumor** | **III** | **1+** | **100%** | **4** |
|  |  |  | **normal** |  | **1+** | **100%** | **4** |
| **63** | **45** | **male** | **tumor** | **III** | **1+** | **100%** | **4** |
|  |  |  | **normal** |  | **1+** | **60%** | **3** |
| **64** | **59** | **male** | **tumor** | **II-III** | **1+** | **100%** | **4** |
|  |  |  | **normal** |  | **2+** | **100%** | **8** |
| **65** | **55** | **male** | **tumor** | **II-III** | **1+** | **100%** | **4** |
|  |  |  | **normal** |  | **1+** | **100%** | **4** |
| **66** | **48** | **male** | **tumor** | **II** | **1+** | **＜1%** | **0** |
|  |  |  | **normal** |  | **2+** | **100%** | **4** |
| **67** | **63** | **male** | **tumor** | **II** | **1+** | **100%** | **4** |
|  |  |  | **normal** |  | **1+** | **100%** | **4** |
| **68** | **59** | **male** | **tumor** | **II** | **1+** | **100%** | **4** |
|  |  |  | **normal** |  | **2+** | **100%** | **8** |
| **69** | **59** | **male** | **tumor** | **II** | **1-2+** | **100%** | **6** |
|  |  |  | **normal** |  | **2+** | **100%** | **8** |
| **70** | **73** | **female** | **tumor** | **II-III** | **1+** | **100%** | **4** |
|  |  |  | **normal** |  | **1+** | **100%** | **4** |
| **71** | **71** | **male** | **tumor** | **II** | **1+** | **100%** | **4** |
|  |  |  | **normal** |  | **1+** | **100%** | **4** |
| **72** | **40** | **female** | **tumor** | **II-III** | **1-2+** | **100%** | **6** |
|  |  |  | **normal** |  | **1+** | **100%** | **4** |
| **73** | **73** | **female** | **tumor** | **II-III** | **1+** | **100%** | **4** |
|  |  |  | **normal** |  | **2+** | **100%** | **8** |
| **74** | **44** | **female** | **tumor** | **II-III** | **2+** | **100%** | **8** |
|  |  |  | **normal** |  | **2+** | **100%** | **8** |
| **75** | **69** | **male** | **tumor** | **III** | **2+** | **100%** | **8** |
|  |  |  | **normal** |  | **1-2+** | **100%** | **6** |

**(1) Staining intensity score: score 0 = no positive staining; score 1 = 1+; score 2 = 2+; score 3 = 3+.**

**(2) Positive-staining score: score 0 = (＜1%) ; score 1 = (1-25%); score 2 = (26-50%); score 3 = (51-75%); score 4 = (76-100%).**

**(3)** **Cumulative score = staining intensity score × positive-staining score.**
