# Supplementary material for: RGS6 suppresses TGF-β-induced epithelial–mesenchymal transition in non-small cell lung cancers via a novel mechanism dependent on its interaction with SMAD4
Source: Cell Death Dis. 2022 Jul 28;13(7):656. doi: 10.1038/s41419-022-05093-0 (PMC9334288; doi:10.1038/s41419-022-05093-0)
Supplement: Supplementary file 7 — Supplmentary Table S4 [file 41419_2022_5093_MOESM7_ESM.docx]

**Table S4. Overall expression of RGS6 in lung cancer and adjacent tissues**

| **Pathological classification** | **RGS6 expression** | | **total** | **χ^2^** | **p value** |
| --- | --- | --- | --- | --- | --- |
|  | **Low** | **High** |  |  |  |
| **Lung cancer** | **56** | **19** | **75** | **14.616** | **＜0.001** |
| **Adjacent tissues** | **33** | **42** | **75** |  |  |
