# Supplementary material for: RGS6 suppresses TGF-β-induced epithelial–mesenchymal transition in non-small cell lung cancers via a novel mechanism dependent on its interaction with SMAD4
Source: Cell Death Dis. 2022 Jul 28;13(7):656. doi: 10.1038/s41419-022-05093-0 (PMC9334288; doi:10.1038/s41419-022-05093-0)
Supplement: Supplementary file 8 — Supplmentary Table S5 [file 41419_2022_5093_MOESM8_ESM.docx]

**Table S5.**  **CRISPR/CAS9 guide RNA sequence**

| **sgRNA name** | **sgRNA sequence** |
| --- | --- |
| **Negative control** | **CGCTTCCGCGGCCCGTTCAA** |
| **RGS6-cas9-1** | **AGATGCAAGATGACAAGACA** |
| **RGS6-cas9-2**  **SMAD4-cas9** | **GGGGAGCCTTATCGCTGCCC**  **CACCGCACTCTCTCCACCTTTGTCTA** |
