# Supplementary material for: RGS6 suppresses TGF-β-induced epithelial–mesenchymal transition in non-small cell lung cancers via a novel mechanism dependent on its interaction with SMAD4
Source: Cell Death Dis. 2022 Jul 28;13(7):656. doi: 10.1038/s41419-022-05093-0 (PMC9334288; doi:10.1038/s41419-022-05093-0)
Supplement: Supplementary file 9 — Supplmentary Table S6 [file 41419_2022_5093_MOESM9_ESM.docx]

**Table S6. Primers for qRT-PCR analysis**

| RGS6 | F: ACTTAGCAAGACTCCAGAGGG  R: GACATCCCAAAAGGCTCGTTC |
| --- | --- |
| SMAD2 | F: CCGACACACCGAGATCCTAAC  R: AGGAGGTGGCGTTTCTGGAAT |
| SMAD3 | F: CATCGAGCCCCAGAGCAATA  R: GTGGTTCATCTGGTGGTCACT |
| SMAD4  Snail  PAI-1 | F: CTCATGTGATCTATGCCCGTC  R: AGGTGATACAACTCGTTCGTAGT  F: CGAAAGGCCTTCAACTGCAAAT  R: ACTGGTACTTCTTGACATCTG  F: TGGTTCTGCCCAAGTTCTCCCTG  R: TGCCACTCTCGTTCACCTCG |
| GAPDH | F: TGCACCACCAACTGCTTAGC  R: GAGGGGCCATCCACAGTCTTC |
|  |  |

^*^ F, forward; R, reverse.

**Table S6.2 Primers for PCR**

| RGS6 | F: CTAGCTAGCTAGGCCACCATGGCTCAAGGATCC  R: GCTACCGGTAGCGGAGGACTGCATCAGGCCCG | NheI  AgeI |
| --- | --- | --- |
| RGS6S | F: CTAGCTAGCTAGGCCACCATGCAAAATAAAGC  R: GCTACCGGTAGCGGAGGACTGCATCAGGCCCG | NheI  AgeI |
| RGS6(△GGL) | F: CTAGCTAGCTAGGCCACCATGGCTCAAGGATCC  R: GCTACCGGTAGCGGAGGACTGCATCAGGCCCG | NheI  AgeI |
| RGS6(△RGS)  SMAD4  SMAD4(△MH1) | F: CTAGCTAGCTAGGCCACCATGGCTCAAGGATCC  R: GCTACCGGTAGC GGGCTCTTTGCTCATCTCTA  F: CTAGCTAGCTAGGCCACCATGGACAATATGTCTA  R: CCCAAGCTTGGGGTCTAAAGGTTGTGGG  F: CTAGCTAGCTAGACCATGATTGATCTCTCAGGAT  R: CCCAAGCTTGGG GTCTAAAGGTTGTGGG | NheI  AgeI  Nhe1  HindIII  Nhe1  HindIII |
| SMAD4(△MH2) | F: CTAGCTAGCTAGGCCACCATGGACAATATGTCTA  R: CCCAAGCTTGGGAGGATGATTGGAAATGGGAG | Nhe1  HindIII |
| RGS6-cas9-1 | F1: TGGGATCTTGTTCCAGGACAT  R2: CACAGATCTTCAGACACCCCTC |  |
| RGS6-cas9-2 | F1: GGTGATGACAGTTGACTTCTGG  R2: TGGTAGGCTCTCAATGACAGC |  |
|  |  |  |

^*^ F, forward; R, reverse.
