# Supplementary figures and images for: RGS6 suppresses TGF-β-induced epithelial–mesenchymal transition in non-small cell lung cancers via a novel mechanism dependent on its interaction with SMAD4
Source: Cell Death Dis. 2022 Jul 28;13(7):656. doi: 10.1038/s41419-022-05093-0 (PMC9334288; doi:10.1038/s41419-022-05093-0)

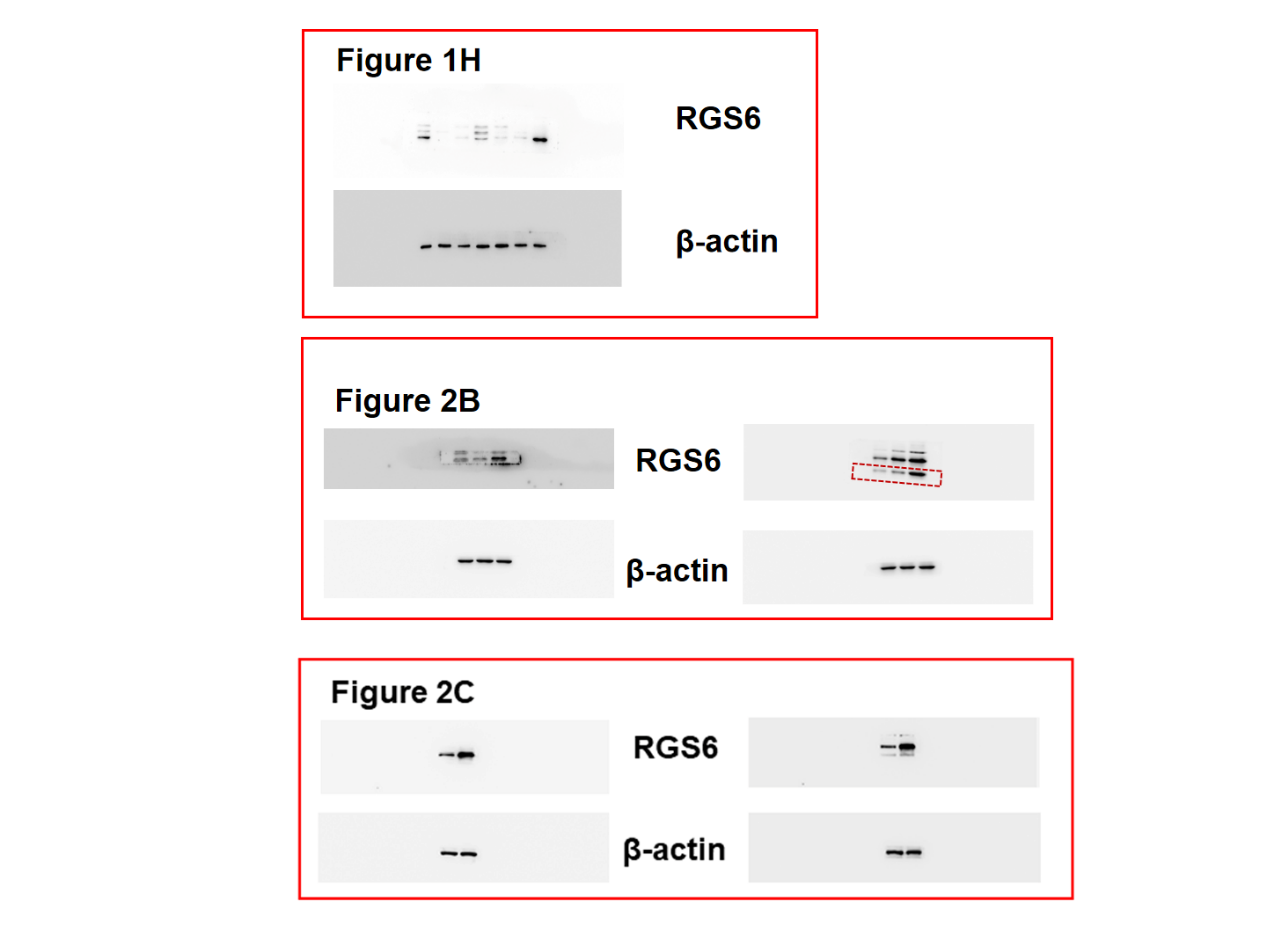


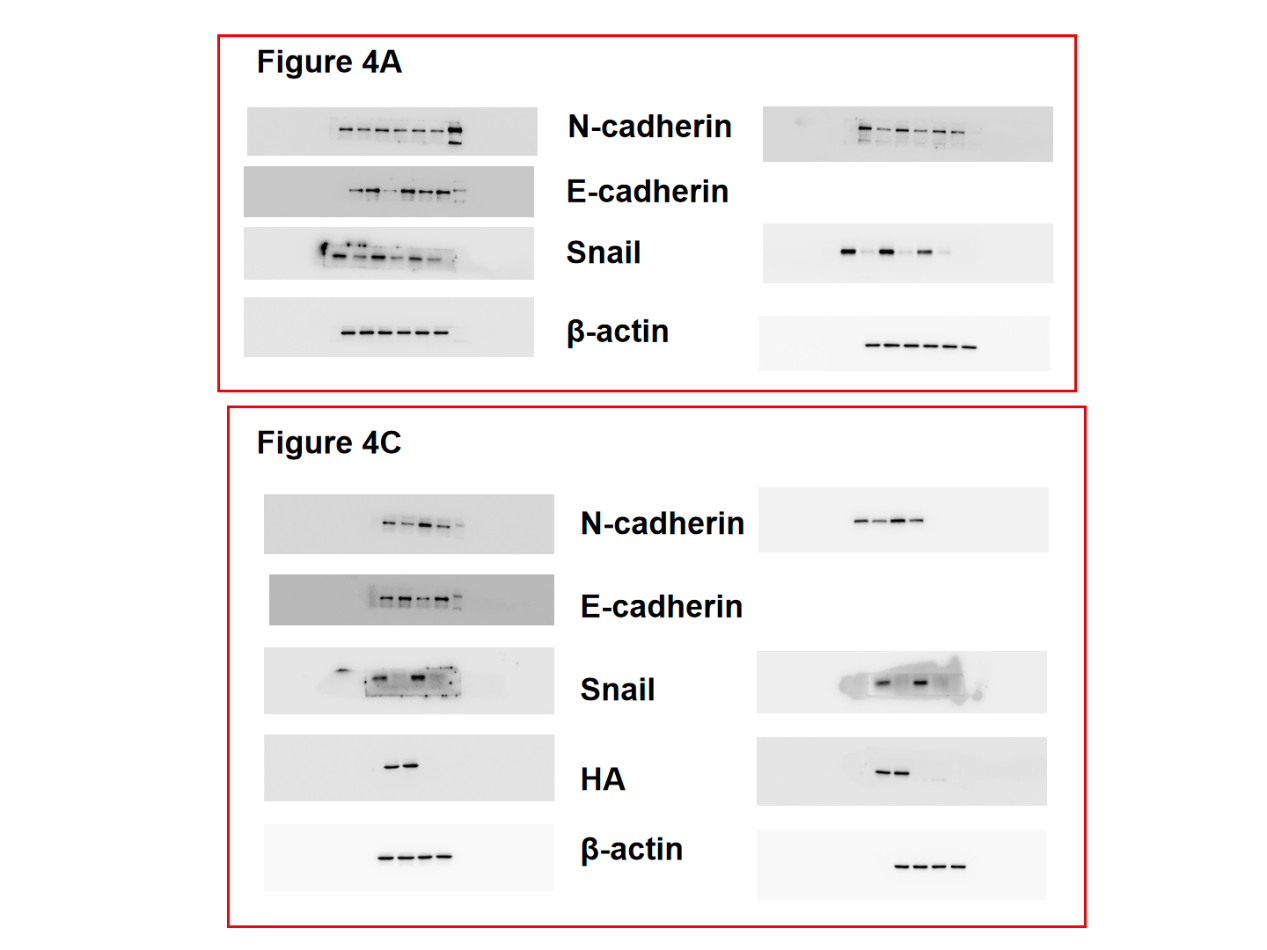


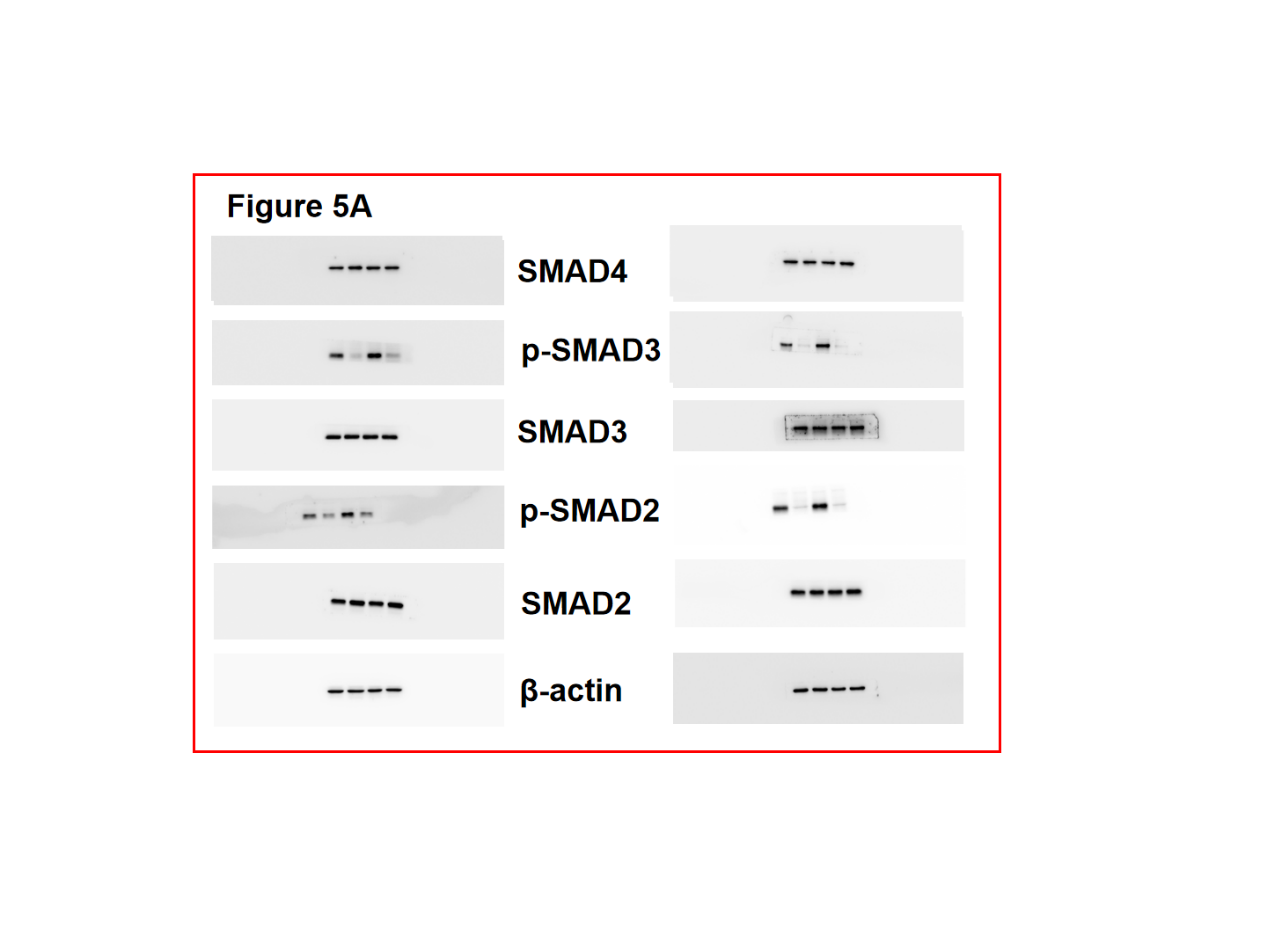


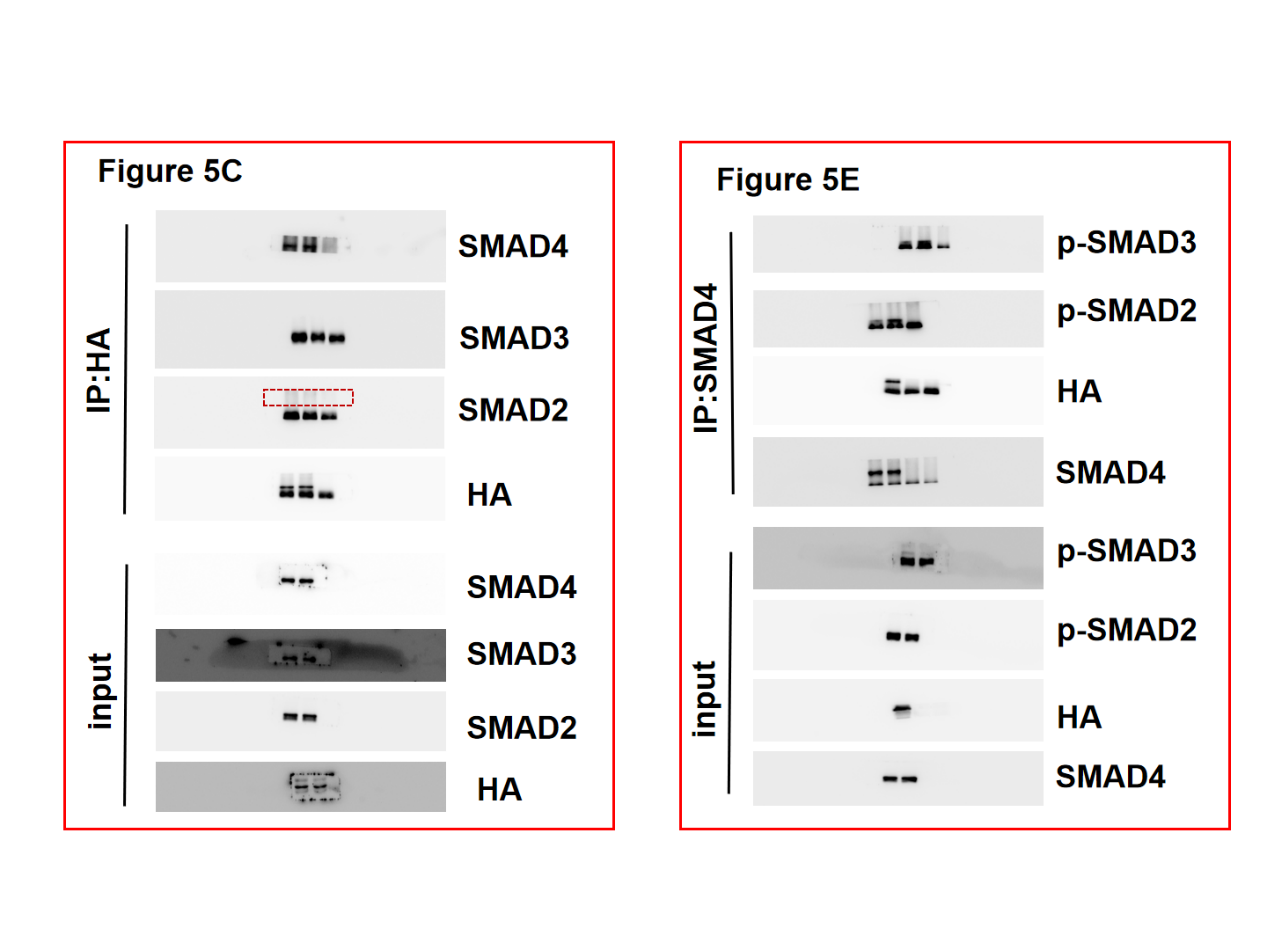


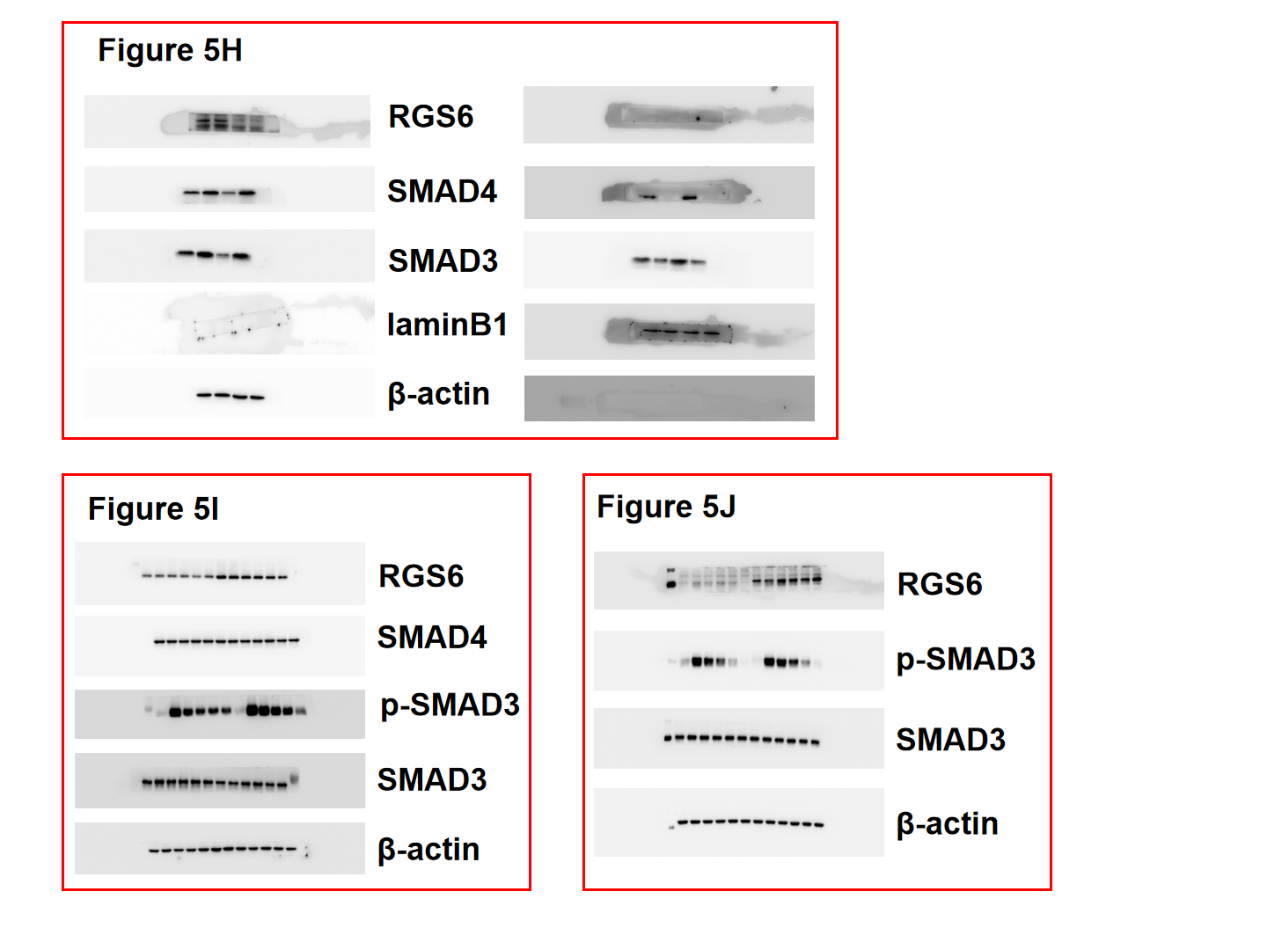


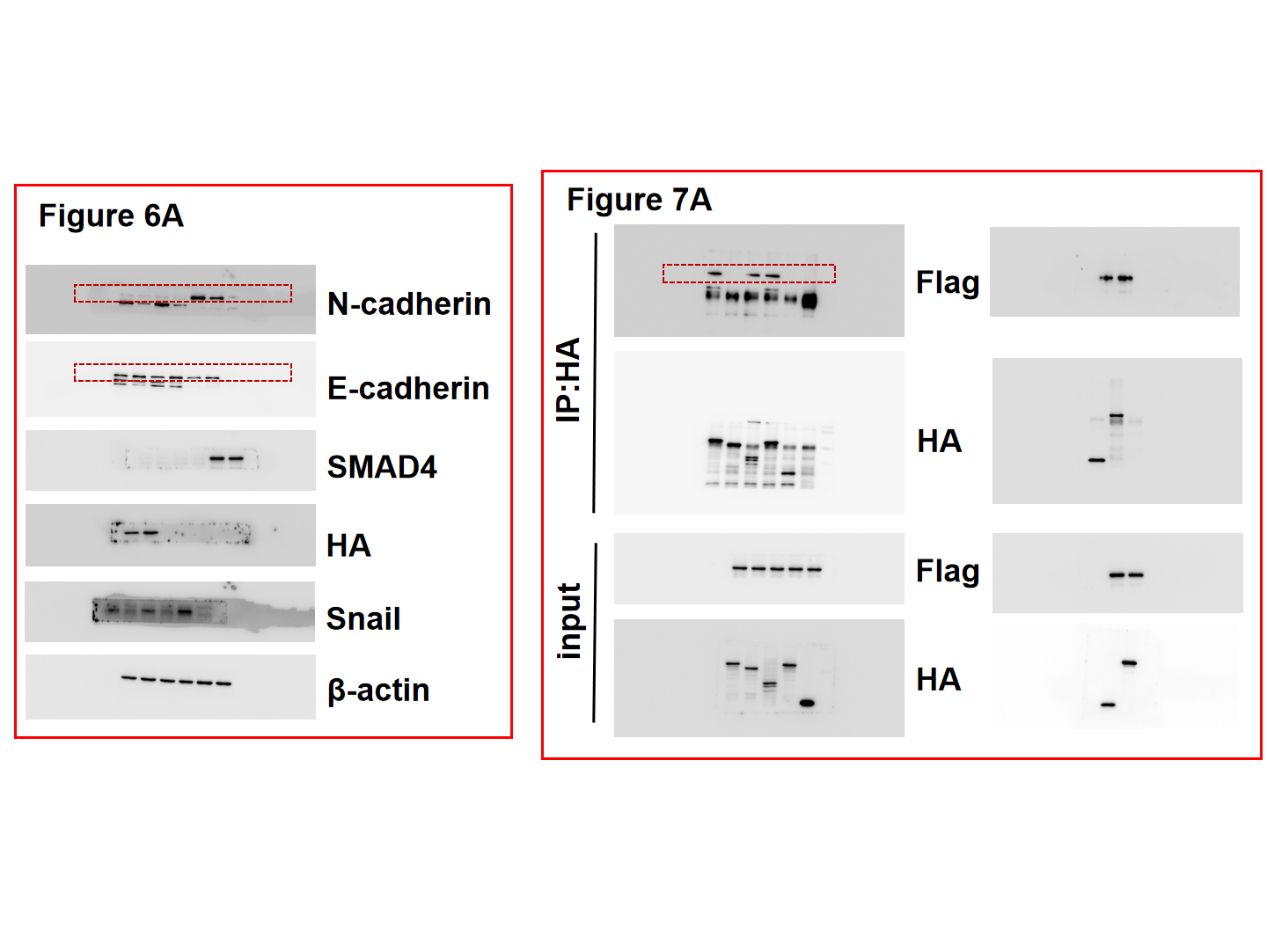


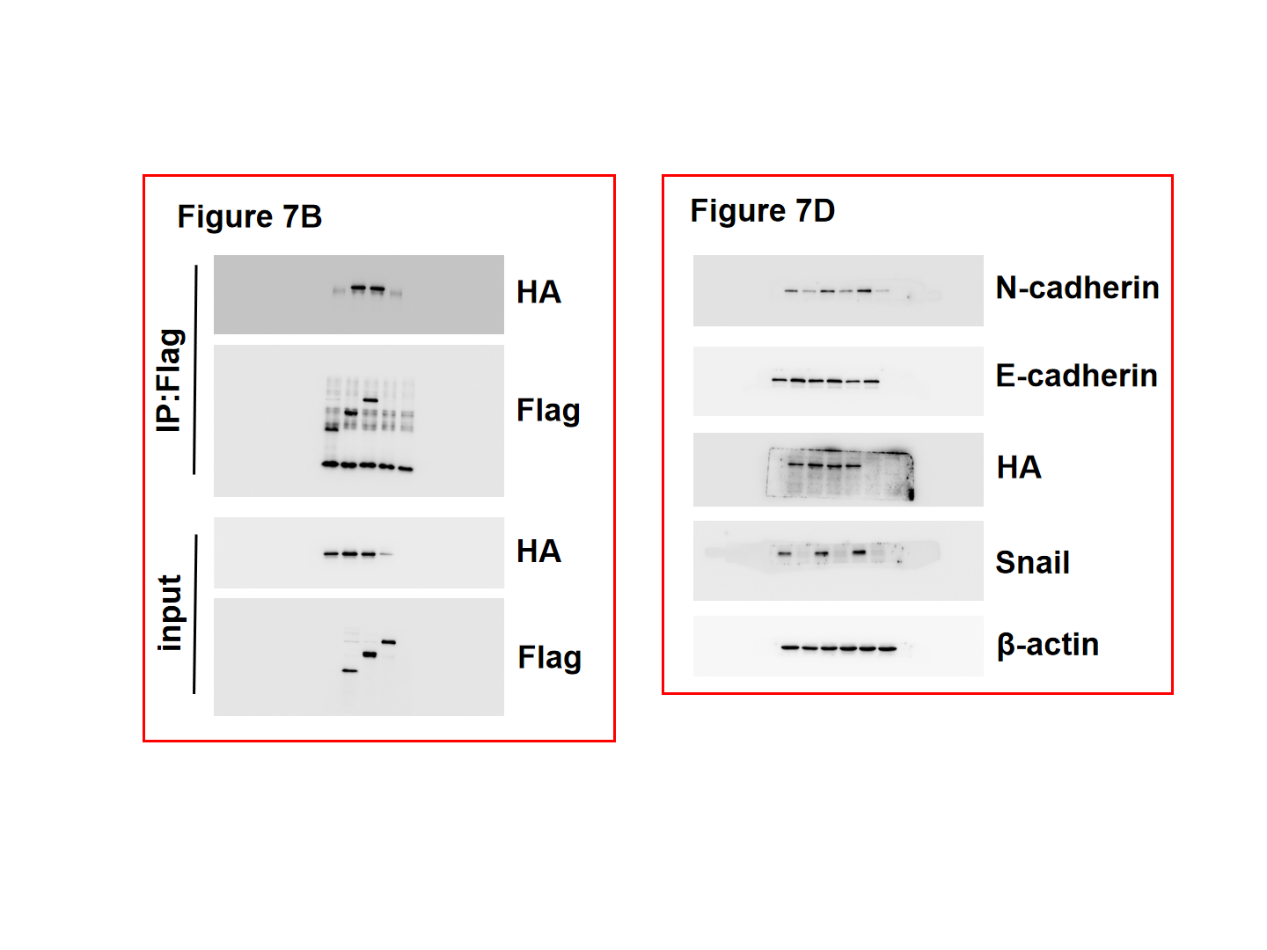


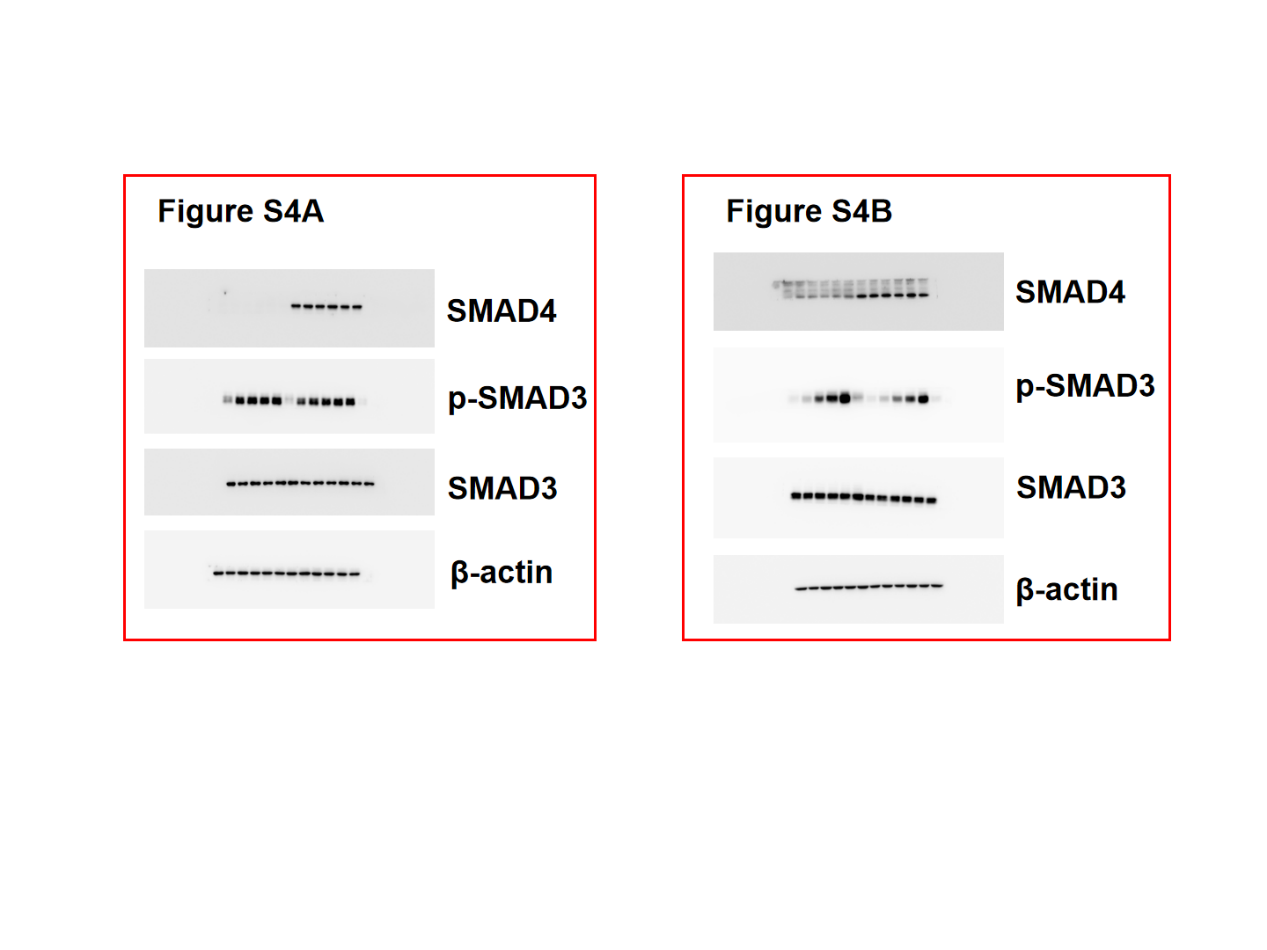


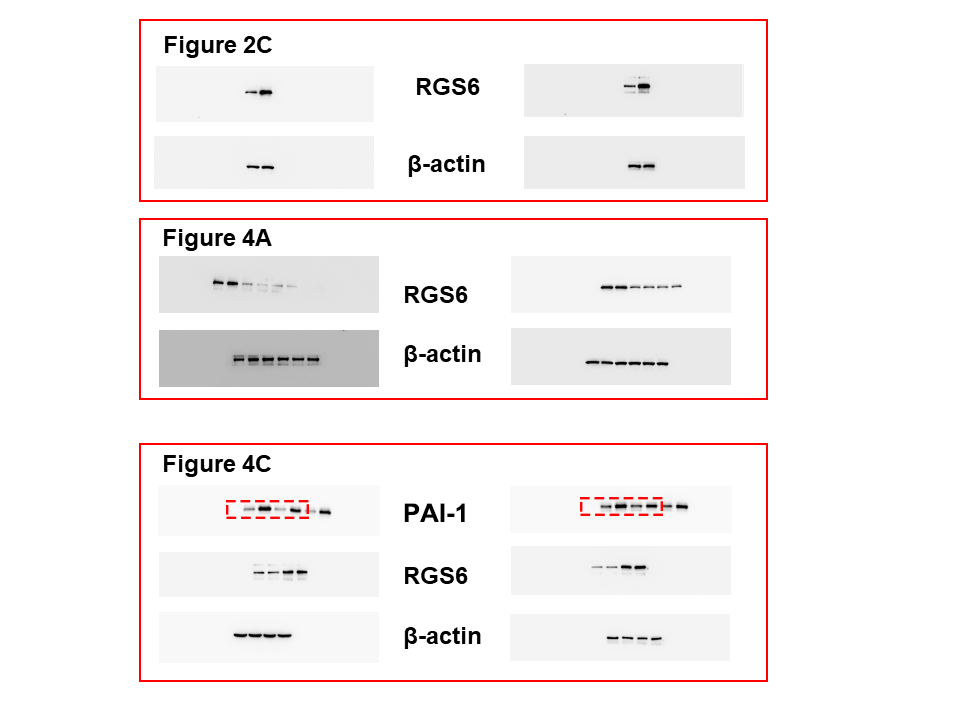


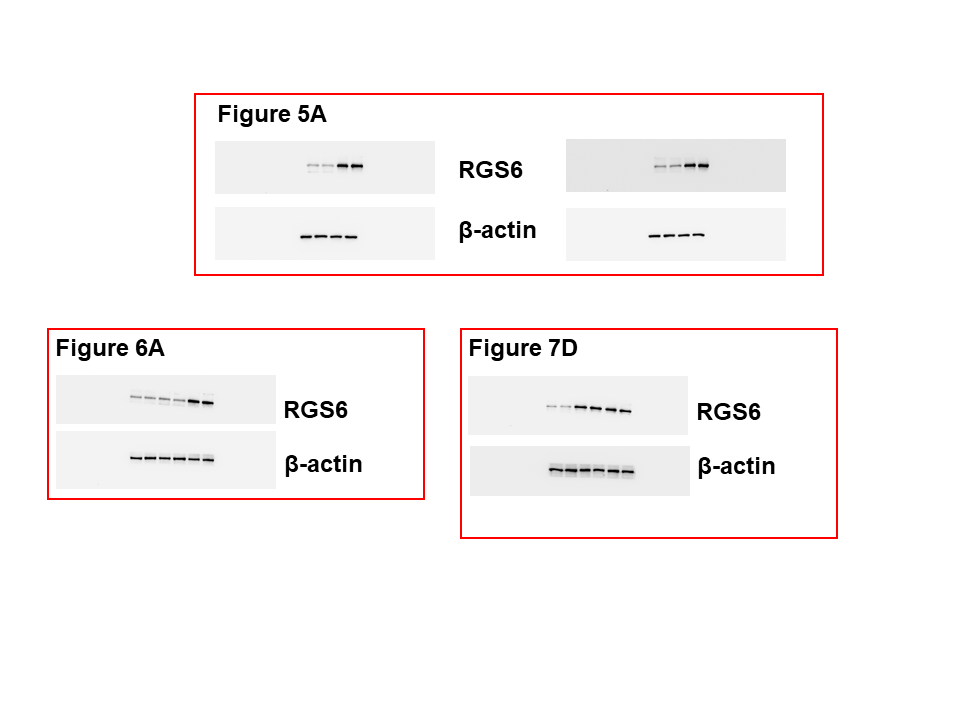

Supplement: Supplementary file 10 — Uncropped WB blots [file 41419_2022_5093_MOESM10_ESM.docx]
